# Postpartum enhancement of spatial learning and cognitive flexibility: an IntelliCage study

**DOI:** 10.1186/s12993-026-00340-2

**Published:** 2026-06-12

**Authors:** Melinda Cservenák, Luca Darai, Tamara Cecília Kállai, Bori Záhonyi, András Lukács, Norbert Bencsik, László Détári, Arpád Dobolyi

**Affiliations:** 1 Laboratory of Molecular and Systems Neurobiology, Department of Physiology and Neurobiology, Eötvös Loránd University, Budapest, 1117 Hungary; 2 Department of Physiology and Neurobiology, Eötvös Loránd University, Budapest, 1117 Hungary

**Keywords:** Mother, Long-term behavior, Cognition, Motivation, Automated monitoring, Home cage

## Abstract

The transition to motherhood has been shown to result in significant changes in the structure and function of the brain, with particular emphasis on the enhancement of cognitive abilities that are essential for survival. Specifically, in murine models, spatial learning and cognitive flexibility have been identified as critical components of mothering. While cognitive enhancements during the postpartum period have been observed in various experimental setups, research using long-term data collection with automated monitoring in a home cage setup is lacking despite its reliability compared to other experimental procedures. This study aims to address this knowledge gap by systematically examining spatial learning and cognitive flexibility in female mice during the reproductive stages using the IntelliCage system. The results showed that female mice in the postpartum phase outperformed pregnant and nulliparous females in place learning, reversal learning, and fixed schedule drinking tasks, demonstrating faster adaptation and superior retention of information. Postpartum mothers showed elevated cognitive ability in spatial learning, and also showed increased flexibility compared to the control/pregnant group suggesting a greater ability to update previously learned associations. The identification of these improvements in maternal behavior may provide novel insights into how reproductive experiences impact brain function, with implications for maternal health and broader cognitive research.

## Introduction

The postpartum period is characterized by profound physiological, emotional, and behavioral transformations aimed at optimizing maternal care for offspring survival [1]. These adaptations are underpinned by extensive neurobiological changes that enhance maternal motivation, sensory processing, and cognitive functioning. Among these adaptations, cognitive flexibility - the ability to adapt behavioral responses to changing environmental contingencies - and spatial learning have emerged as critical components of successful caregiving [44]. These cognitive processes enable mothers to navigate their environment efficiently, locate resources, and respond effectively to the dynamic needs of their offspring. The study of cognitive flexibility has been relatively limited in mothers, despite the likelihood that these functions are critical for maternal responsiveness and parenting quality in both rodents and humans [2, 28]. Thus, the specific mechanisms and extent of behavioral improvements during the postpartum period remain incompletely determined and understood. The IntelliCage system offers a unique platform to investigate these cognitive changes in rodents under semi-naturalistic and socially enriched conditions [25]. This automated system enables the monitoring of multiple animals simultaneously and provides a comprehensive analysis of their behavior across various cognitive tasks [29]. By allowing precise tracking of individual animals, IntelliCage eliminates the need for human intervention, thereby minimizing stress and enabling the assessment of spontaneous behavior. It also facilitates the implementation of sophisticated behavioral paradigms that probe different dimensions of cognition, including spatial learning, reward-based motivation, and cognitive flexibility [4], particularly through paradigms such as place learning and reversal learning [45]. Place learning tasks assess an animal’s ability to associate a specific location with a reward [12], while reversal learning evaluates cognitive flexibility by requiring animals to adapt to a new reward location [21]. These paradigms are particularly relevant for studying maternal cognitive adaptations, as they reflect the types of challenges mothers face in the wild, such as finding food, avoiding predators, and managing the competing demands of caregiving and self-maintenance.

Although cognitive enhancements during the postpartum period have been documented in other experimental setups [8, 16, 31], no studies have systematically examined these changes in the context of the IntelliCage system. Moreover, existing studies often focused on specific behavioral aspects of maternal adaptations, with limited integration of them. Addressing this gap, the present study aims to provide a comprehensive analysis of cognitive flexibility and spatial learning in postpartum females using IntelliCage.

The present study employed a sequence of behavioral tasks designed to assess multiple dimensions of cognition and motivation across reproductive states: pregnancy, postpartum, and control (non-pregnant) conditions. During the adaptation phases, animals were introduced to the IntelliCage environment and trained to perform nosepoke behaviors to access water. Following this, place learning and reversal learning tasks were conducted to evaluate spatial learning and cognitive flexibility. These tasks required animals to associate specific corners with water availability and adapt to changes in reward contingencies. Finally, fixed schedule drinking tasks were implemented to examine anticipatory and persistent behaviors in response to restricted water access.

## Methods

### Animals

This study was performed in line with the principles of the Declaration of Helsinki. Approval was granted by the Workplace Animal Welfare Committee at Eötvös Loránd University and the National Scientific Ethical Committee on Animal Experimentation (Date: 29.06.2021/No: PE/EA/568-7/2021). The research involving mice was conducted in accordance with the standards set forth by the Animal Hygiene and Food Control Department of the Ministry of Agriculture in Hungary (40/2013), which are consistent with the EU Directive 2010/63/EU for animal experiments. The animals were housed in accordance with standard laboratory conditions, maintaining a temperature of 23 ± 1 °C and humidity levels between 50 and 60% both when being in the IntelliCage, and before and after that. They experienced a 12-hour light/dark cycle, with the lights turning on at 5:00 AM. They were provided with unlimited access to food (SM R/M-H, 1534-00; Ssniff, Soest, Germany) and drinking water.

Overall, 44 female C57BJ/6J mice were used for this study. At 2–3 months of age, prior to IntelliCage testing, a radiofrequency identification transponder chip was subcutaneously implanted into the dorso-cervical region under isoflurane inhalation anesthesia. Mice were allowed 1 week to recover and were then introduced into the IntelliCage. The animals were always implanted with RFID chips one week before being placed in the IntelliCage. The start of pregnancy was counted back from the time of birth. The status of each animal was monitored during weekly litterings and daily system checks using the unique RFID chips allowing the animals to be identified individually. The condition of the mothers’ nipples was assessed to determine sucking.

### IntelliCage setup

**Fig. 1:**
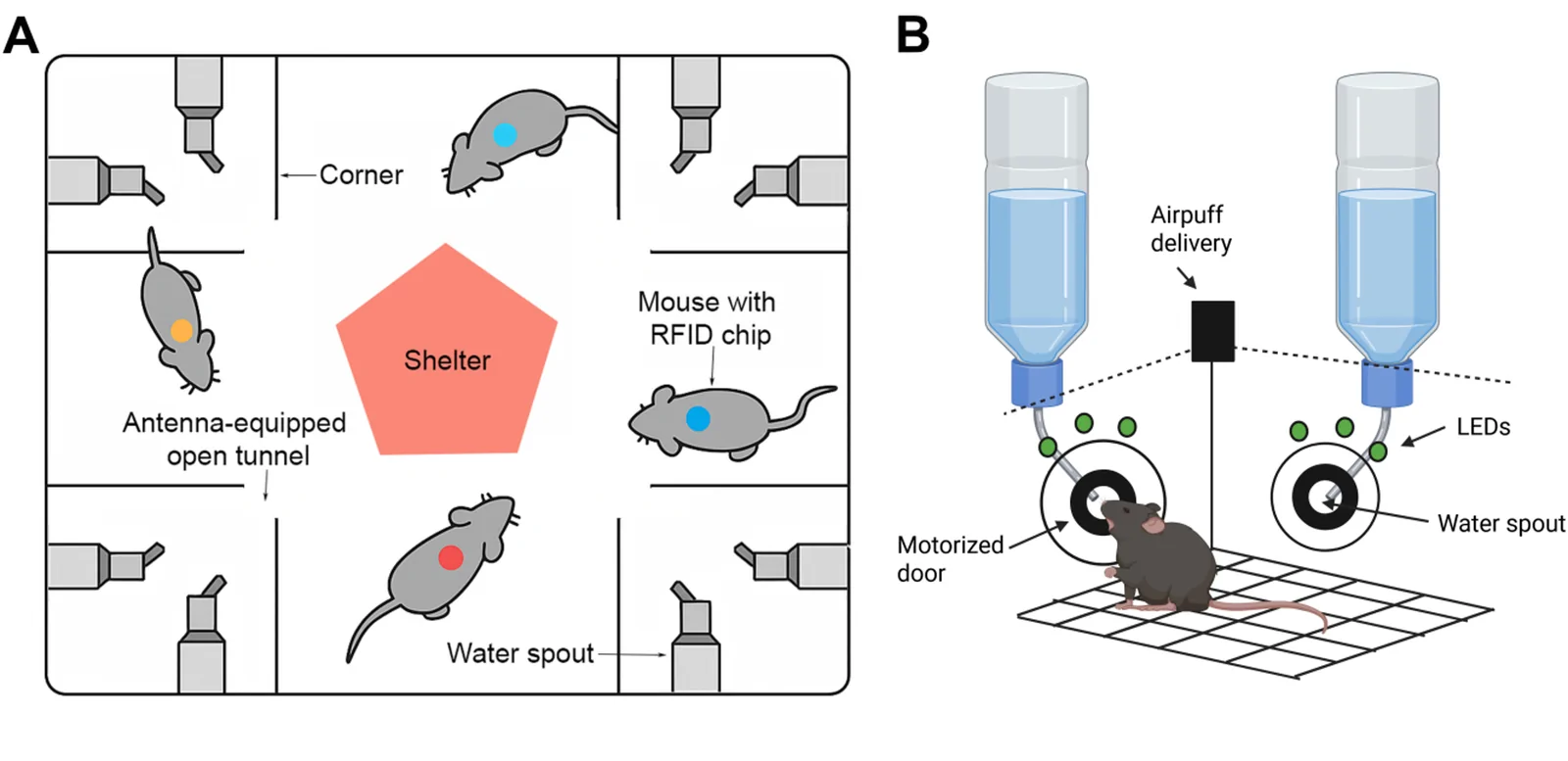
Schematic illustration of the IntelliCage system. **A** IntelliCage contains four corners that are programmed to access water based on nosepokes. **B** Each corner of the IntelliCage is equipped with two motorized doors, LEDs above doors and an air puff delivery nozzle. Mice are not to scale in the figure. Panels of the figure were created in https://BioRender.com

The IntelliCage is an automated behavioral testing system designed for use with laboratory rodents in a group-housed and home-cage environment. It allows researchers to monitor and manipulate the behavior of animals without human interference, which helps reduce stress and increase the validity of behavioral data. It enables high-throughput and long-term monitoring by continuous 24/7 data collection over days or weeks. The key benefit is that it eliminates subjective bias, thereby improving the reproducibility of the data. The IntelliCage apparatus, with dimensions of 39 cm × 58 cm × 21 cm, comprised of four accessible corners, accessible via an antenna-equipped tunnel (Fig. 1). Scanners situated at each corner entrance were programmed to detect the entry of each animal by reading the previously implanted RFID. The sensors located on the nose port and waterspout, respectively, were employed to detect instances of nosepoking and licking. The mice were provided with unlimited standard mouse chow in the middle compartment of IntelliCage throughout the entire experiment.

### Data acquisition and visualization

The following data was recorded and obtained from the IntelliCage system: the number of visits, nosepokes and licks made by all animals in all corners. The data obtained from the IntelliCage system was subsequently visualised using bespoke software written in VBA (Visual Basic for Applications) within Microsoft Excel. The software was designed to directly read text files exported from IntelliCage, namely those pertaining to the environment, visits and nosepokes. Analytical data is derived from the raw data at varying resolutions (1, 2, 4, 6, or 12 h) across a comprehensive range of variables, including Correct Visits, Visits without Licks, and so forth. This approach allows for the capture of both the frequency and duration of events. The analytical data can be displayed in two plots, each comprising two graphs. For each plot, the user is able to select the desired time resolution, the point of origin, and the duration of the time period in question. The graphs can display data for individual animals or a chosen average. For averaging, animals can be individually selected (e.g. by sex), and the start time for each animal can be set individually, allowing synchronisation with the date of parturition, for example. The analytical data displayed on the graphs is also available in table form to facilitate export to external statistical or graphical software. For averages, values for sumX, sumX2, n, and SEM are also included to support further statistical analysis.

### Behavioral tasks

**Fig. 2:**
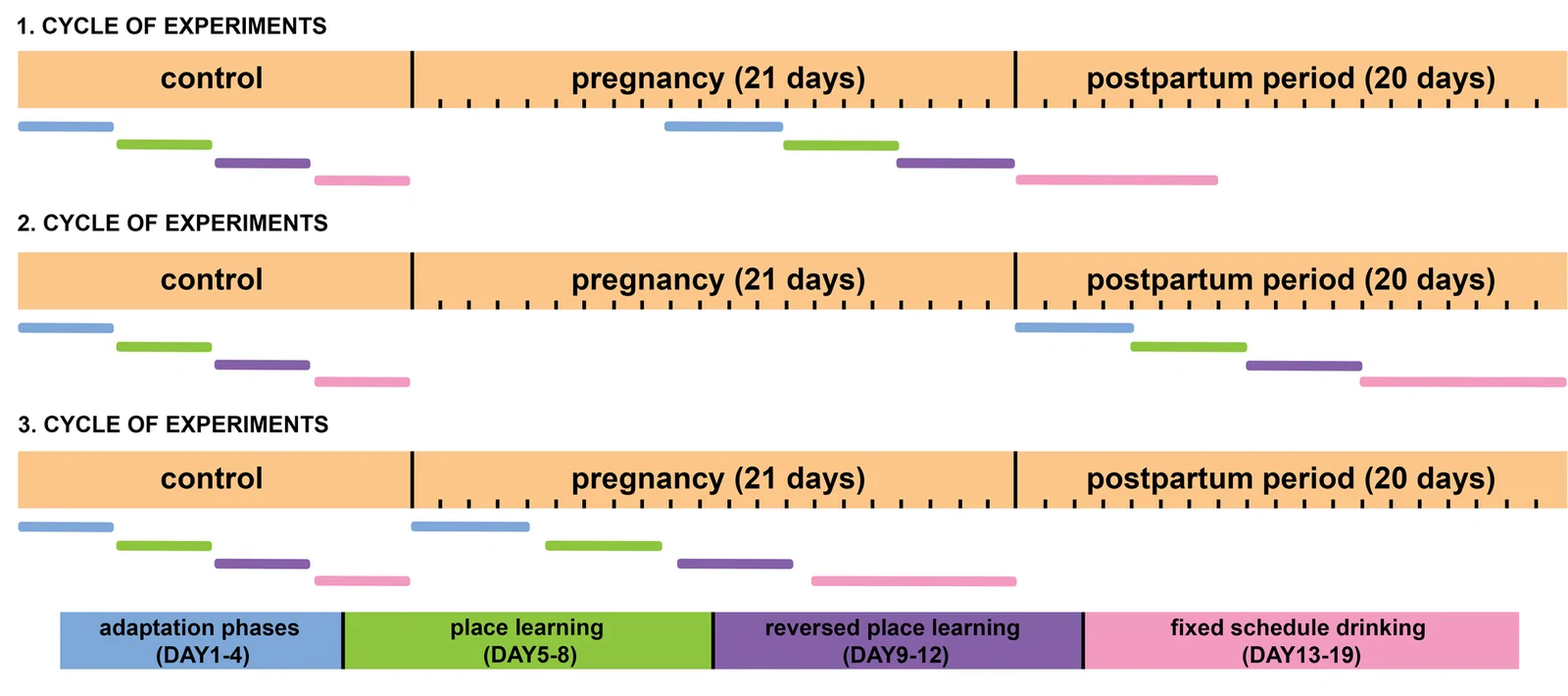
Behavioral battery timeline of the 3 cycles of experiments. The different colored lines indicate the individual tasks and their positioning indicates the start and duration of the task according to the reproduction cycle. The colors represent the different tasks defined in the bottom of the figure

The sequence of behavioral tasks in the IntelliCage was as follows: (1) Adaptation, consisting of free adaptation (1 day) and nosepoke adaptation phases (3 days); (2) Place learning (4 days) and reversed place learning (4 days); and (3) Fixed schedule drinking (7 days) (Fig. 2). Overall data come from a total of 3 cycles of experiments. In the first cycle, the number of control (non-mother) animals was 5 and the number of pregnant females was 11. In the first cycle, we mated the females before they entered the IntelliCage (they were with the males for 5 days to ensure that they were in estrus at least once), so they gave birth at the beginning of the fixed schedule drinking task or in the first days of this task and the control animals were those that did not become pregnant. So in the first cycle, the animals were already mothers in the fixed schedule drinking task. In the second cycle, 12 mice participated (*n* = 5 for control, and 7 for mother group, respectively). In the second cycle, females gave birth before the experiments (either immediately before or 1–2 days before). In the third cycle, female mice were mated after the nosepoke adaptation and have completed the learning tasks during pregnancy. In this cycle, 4 control animals and 12 pregnant animals completed the tasks. So the order of testing was exactly the same in the three cycles, only the time of mating differed.

We also present the number of animals in a different way based on their participation in the various phases. During the adaptation phase, the number of mice in each group was as follows: control: *n* = 9 (this number was accumulated over two cycles, Cycle 1 and Cycle 2), pregnant: *n* = 11 (this number of animals was recorded during the Cycle 1), mother: *n* = 6 (Cycle 2). During the place learning phase, the number of animals in each group was as follows: control: *n* = 8 (Cycle 2 and Cycle 3), pregnant: *n* = 12 (Cycle 3), mother: *n* = 8 (Cycle 2). During the reversed place learning phase, the number of mice in each group was as follows: control: *n* = 8 (Cycle 2 and Cycle 3), pregnant: *n* = 12 (Cycle 3), mother: *n* = 8 (Cycle 2). During the fixed schedule drinking phase, the number of animals in each group was as follows: control: *n* = 9 (Cycle 1 and Cycle 3), pregnant: *n* = 12 (Cycle 3), mother: *n* = 11 (Cycle 1).

All pregnant and mother were in their first reproductive experience. We chose primiparous animals for our experiments because a previous paper on rats described primiparous rats as performing better than multiparous and nulliparous rats in hippocampus-dependent learning tasks [37].

#### Adaptation phases

Free adaptation:

During the first day, all doors were left open, allowing the mice unrestricted access to the water bottles, helping them acclimate to the new environment. The following metrics were calculated for this phase for each day: total visits, total nosepokes, total licks, average lick number per nosepoke, visits with nosepoke% (the percentage of visits with nosepoke divided by the number of total visits), visits with lick % (the percentage of visits with lick divided by the number of total visits).

Nosepoke adaptation:

In the next three days, the doors remained closed and could be opened only with a nosepoke in a corner, training the animals to nosepoke to access water. The same metrics were calculated as for free adaptation.

#### Place learning and reversed place learning

Place learning phase:

For the four days duration of the place learning phase, water was available exclusively in only one of the four corners for each mouse, the designated reward corner (or correct corner). This specific corner for each mouse was chosen based on their previous visit habits, selecting from the least visited corners to avoid any inherent corner bias. To prevent overcrowding and learning by imitation, the designated reward corners were balanced by the number of mice, with an upper limit of four mice per corner. Besides measuring total visits, total licks, and total nosepokes (to assess appetitive drive, that is to seek place for drinking) per day, the average number of lick per correct nosepoke (to assess the drive of the consummatory behavior, that is drinking), and a parameter called „correct%” was also calculated for this task: (Number of visits to the correct corner with a nosepoke)/(Total corner visits) to measure the ability of the animals to find their designated reward corner. Since baseline visits of the correct corners varied between animals before place learning started, another parameter called Preference% was introduced in place learning phase to calculate the difference in correct% measured between the days of place learning and the final day of nosepoke adaptation. In addition, the speed of learning between day 1 and day 2 was measured as the slope of the learning curve.

Reversed place learning phase:

For the following four days, water was available only in the opposite corner (reversal). The same parameters were recorded as for the place learning phase. Two additional parameters were also calculated in this phase: perseveration% is the number of visits in the corner that was marked as correct in the place learning phase divided by the total number of visits. Decrease in preference% during reverse place learning was calculated as the difference in correct% between the last day of place learning phase and the first day of reversed place learning phase for the new correct corner.

#### Fixed schedule drinking

For the subsequent seven days, water access was restricted to two 1-hour periods during the light phase (starting at 10 AM and 3 PM). The animals’ access to water was limited only in time, not in space, i.e. they could drink in all four corners. Mice still had to nosepoke to open access to the spout, but any nosepoke outside these periods did not grant access to the water. The behavior of the mice was analyzed 1 h before, during, and 1 h after water access to assess anticipatory and persistent behaviors. The following metrics were calculated in this phase: total visits, total nosepokes, total licks, and average lick number per nosepoke (the latter two are calculated only during water access).

### Statistical analyses

The IntelliCage data were analyzed using 1-way and 2-way ANOVA with repeated measures when applicable, employing GraphPad Prism 8.1.2. One factor was the group of animals, the other if applied, was the time (4 h or days). When significant changes were found, Bonferroni’s corrected post hoc tests were conducted. A significance level of *p* < 0.05 was used. Normality was tested with D’Agostino-Pearson normality test and Shapiro-Wilk normality test, while equal variance was tested with Bartlett’s test (if data were normal) and Brown-Forsythe test (when data may not be normally distributed). When conditions did not allow for ANOVA, the Kruskal-Wallis test was used instead. The Pearson correlation coefficient (r) was used as a measure of effect size.

## Results

### Adaptation phase

**Fig. 3:**
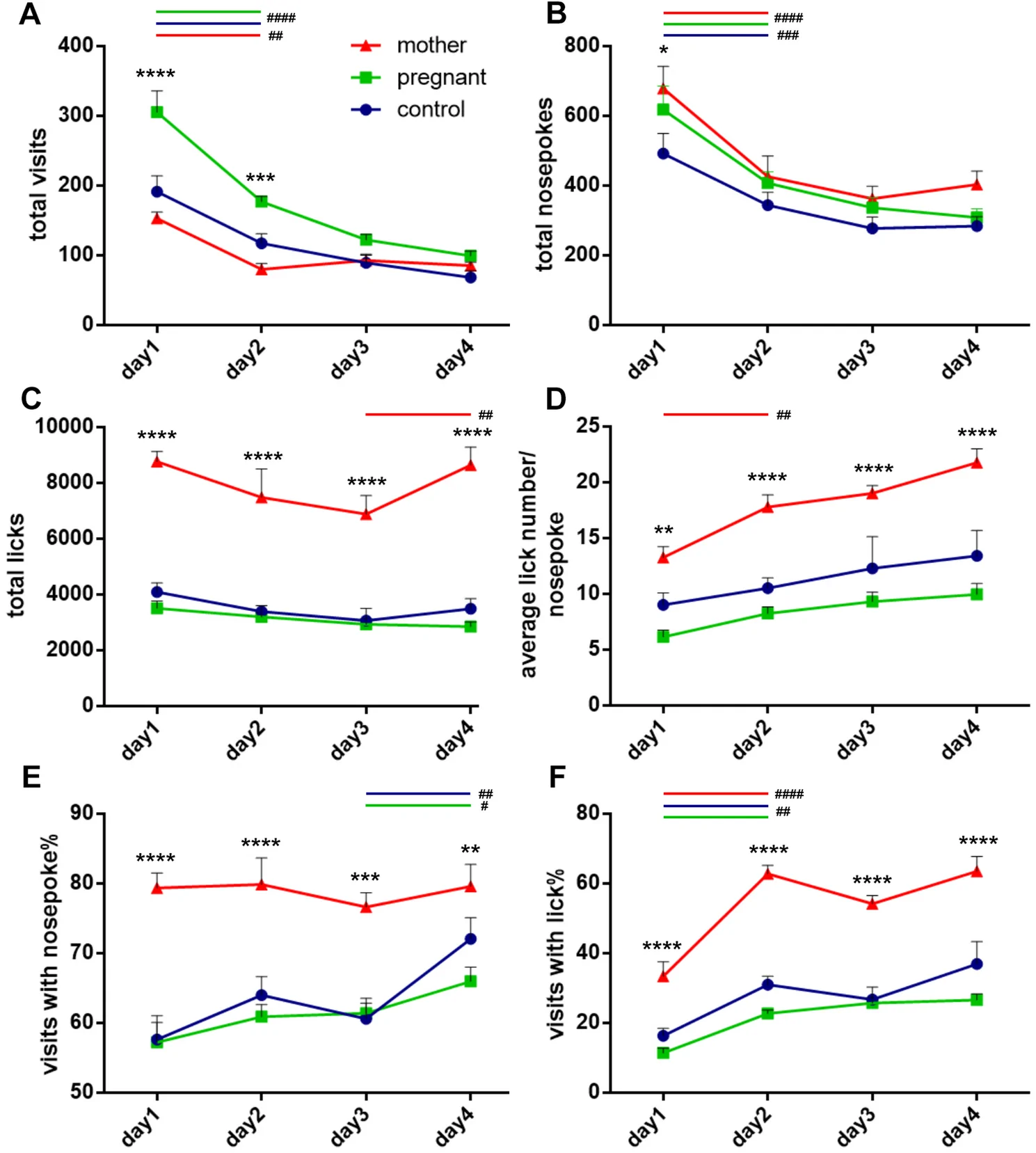
Adaptation phase. Throughout the 4-day adaptation period, mice were able to move freely into any of the four corners and had to learn to nosepoke to access water. Nosepoke adaptation phase started on 2nd day, when animals had limited time (5 s) to nosepoke. **A** We found that pregnant mice made more corner visits on day 1 and 2 compared to mother and control mice. We also found that the number of visits decreased from day 1–4 in all groups. **B** Total nosepokes, a parameter indicative of appetitive motivation, also decreases as the days progress in all three groups. **C** Significant differences were detected in the number of total licks (water consumption) throughout the entire period between mother mice and pregnant/control mice. **D** Average lick number per nosepoke is significantly higher in the group of mothers, suggesting a difference in consummatory behavior. **E** From day 1, significant differences in visits with nosepoke% (indicative for learning) were observed between mother and pregnant/control mice. **F** We found that mother mice made more total visits with lick than pregnant and control mice from day 1 of adaptation suggesting elevated water-seeking motivation in this group. Data are presented as means +/- SEM. **p < 0.01, ***p < 0.001, ****p < 0.0001

Before facing any challenge, it is crucial to assess the animals’ baseline activity and their responses to the new environment. Furthermore, the degree and speed of adaptation provides information on the animals’ motivation and cognition. During the adaptation, the number of total visits decreased across days in all groups (Fig. 3A) interpreted as decreased exploratory activity. We found a significant reproductive status by day interaction; post hoc analysis revealed that pregnant mice made more corner visits than control and mother mice on day 1. The number of total nosepokes also decreased over time (Fig. 3B). Specifically, a significant decrease in total visits and total nosepokes was observed between day 1 and day 2 in all three groups, which did not show a significant decrease afterwards, indicating that all groups had adapted to the new environment during the 4 days (Fig. 3A, B).

Significant differences in total licks were found between the groups (Fig. 3C), indicating that water consumption was higher in postpartum than in control/pregnant females. From day 1, significant differences in visits with nosepoke% were also measured between mother and control/pregnant mice (Fig. 3D), indicating faster learning, that is better learning abilities for postpartum mothers. Furthermore, the mother’s corner visits included drinking behavior in approximately 40% in the first day and 60% thereafter illustrating increased water-seeking motivation. Visits with lick% was significantly lower in control/pregnant than in postpartum mice (Fig. 3E).

Mother mice performed significantly greater numbers of licks per nosepoke, indicating increased consummatory behavior during the whole adaptation period (Fig. 3F).

There was also a significant increase in the average number of licks per nosepoke between day 1 and day 2 in mothers (Fig. 3D). The visits with lick% also increased significantly between day 1 and day 2, which indicates that the animals in all three groups were also accustomed to having access to drinking water in the corners (Fig. 3F). The statistical data for the adaptation phase are shown in Table 1.

**Table 1:**
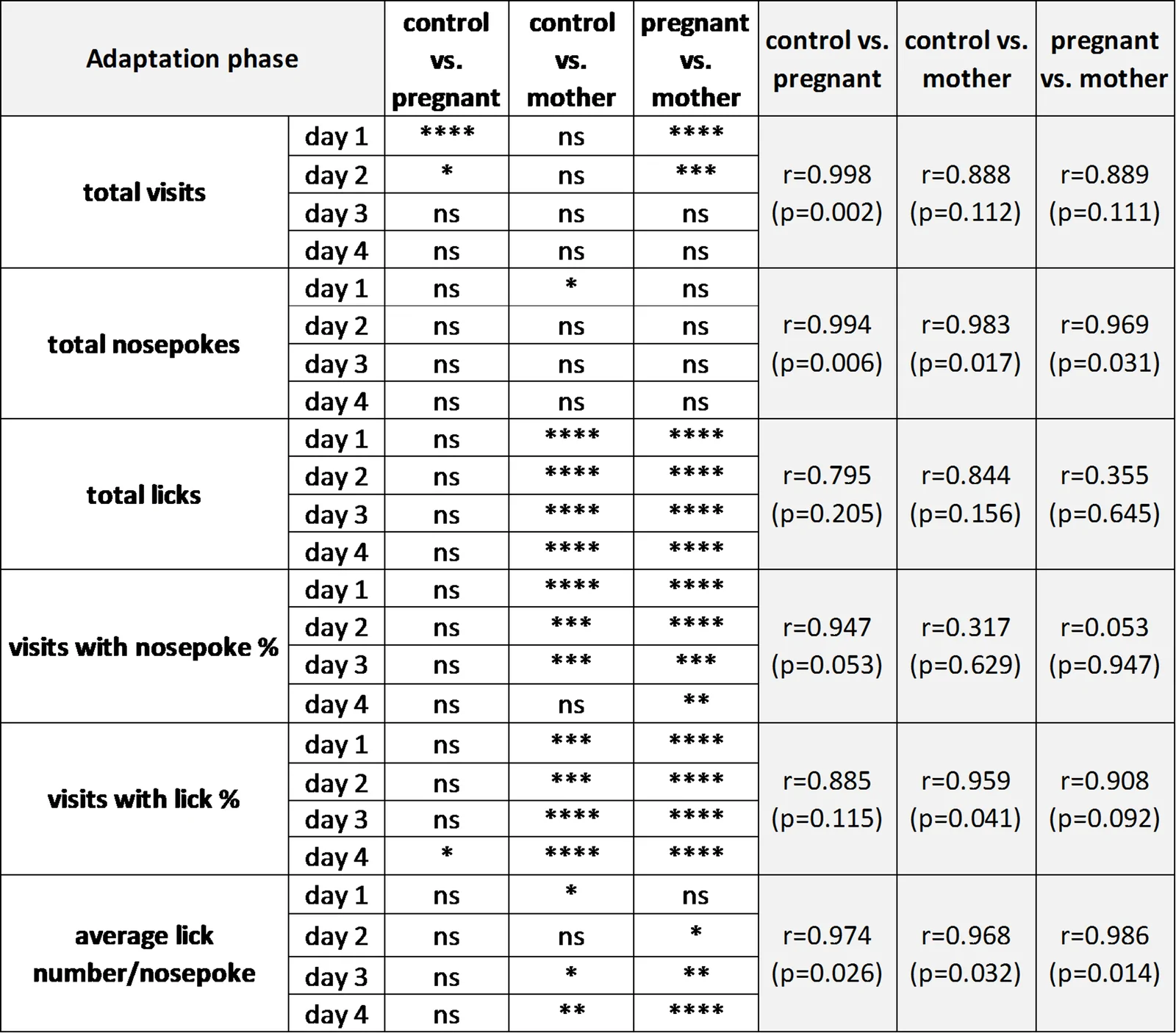
Adaptation phase. Table shows the results of the statistical analysis. Pearson correlation coefficients (r) and their corresponding significance values (p) are shown in a grey background. *p < 0.05, **p < 0.01, ***p < 0.001, ****p < 0.0001

### Place learning

**Fig. 4:**
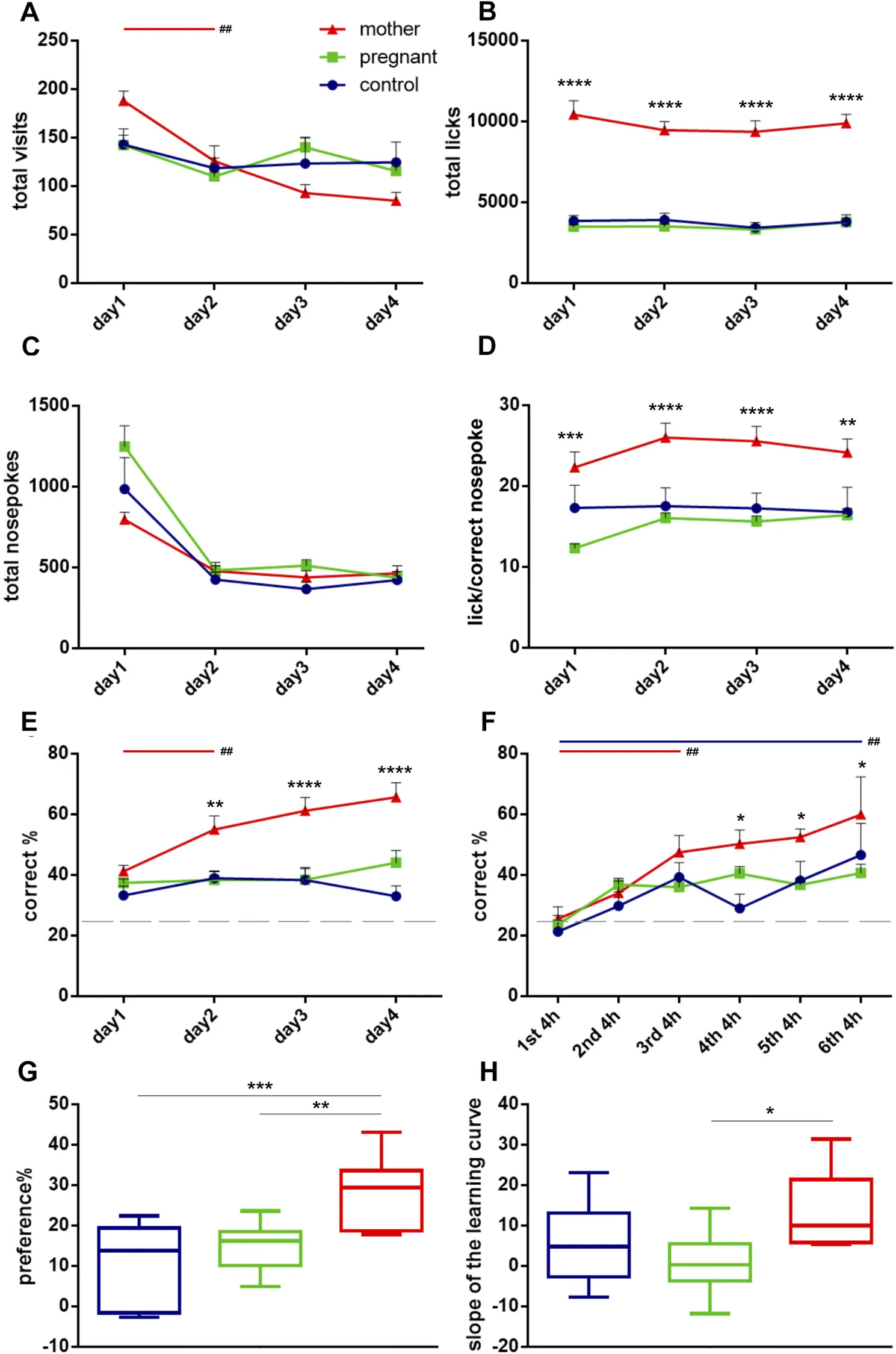
Place learning phase. During the 4-day place learning phase, each animal was assigned to a specific corner where water was available. The other corners were considered incorrect and did not provide access to water. **A** Only on day 3, we found a significant difference in total visits between mother (in mid-postpartum) and pregnant (in early pregnancy) mice. **B** Significant differences in total licks across the 4 days were detected between mother and control/pregnant mice. **C** There was no difference in the total nosepokes between the groups. **D** The average lick/correct nosepoke was significantly higher in mothers. **E** We found a significant effect of day for correct%, illustrating learning across the 4 days, moreover, significant differences were detected from day 2 between mother and control mice. **F **Significant differences between mothers and control/pregnant mice are already observed in the fourth 4 h of the day 1 in correct%. **G **Significant differences were detected in preference % between mother and control/pregnant mice. Data are presented as means +/- SEM. **H** The slope of the learning curve of the mothers between day 1 and day 2 also differed significantly from that of pregnants. *p < 0.05, **p < 0.01, ***p < 0.001, ****p < 0.0001

During place learning phase, we found no significant differences in total visits except a decrease in postpartum mothers from an elevated first day to the second day (Fig. 4A). Similar to the adaptation phase, number of total licks was significantly higher in postpartum mothers than in control/pregnant females and mothers during the entire duration of the learning phase (Fig. 4B). We found a significant increase in correct% in the place learning phase between days 1 and 2 in postpartum mothers but not in other groups (Fig. 4C), suggesting improved learning performance in postpartum mothers. We also established a main effect of reproductive status where mother mice had a significantly higher correct% than control and pregnant female mice from day 2 to day 4. We examined the learning performance of the groups on the first day at a resolution of 4 h and found that significant differences in correct% are already present from the second half of the first day (Fig. 4D). The percentage of correct corner visits (correct%) measured on the first day was already significantly increased between 1 st and 3rd 4 h in mothers, while it was significantly increased between 1 st and 6th 4 h in control group (Fig. 4D). Preference% was determined as the difference in correct% measured between the final day of nosepoke adaptation (when mice could still drink from all four corners) and the initial day of place learning (when water was available only in the correct corner). While an increase was found for all groups suggesting learning, we also found significant differences between groups in this parameter as postpartum mothers has higher value implying they learned faster (Fig. 4D). Figure 4F shows the slope of the correct% curve between day 1 and day 2, a value also significantly higher in postpartum mothers further supporting their elevated learning abilities.

The statistical data for the place learning task are shown in Table 2.

**Table 2:**
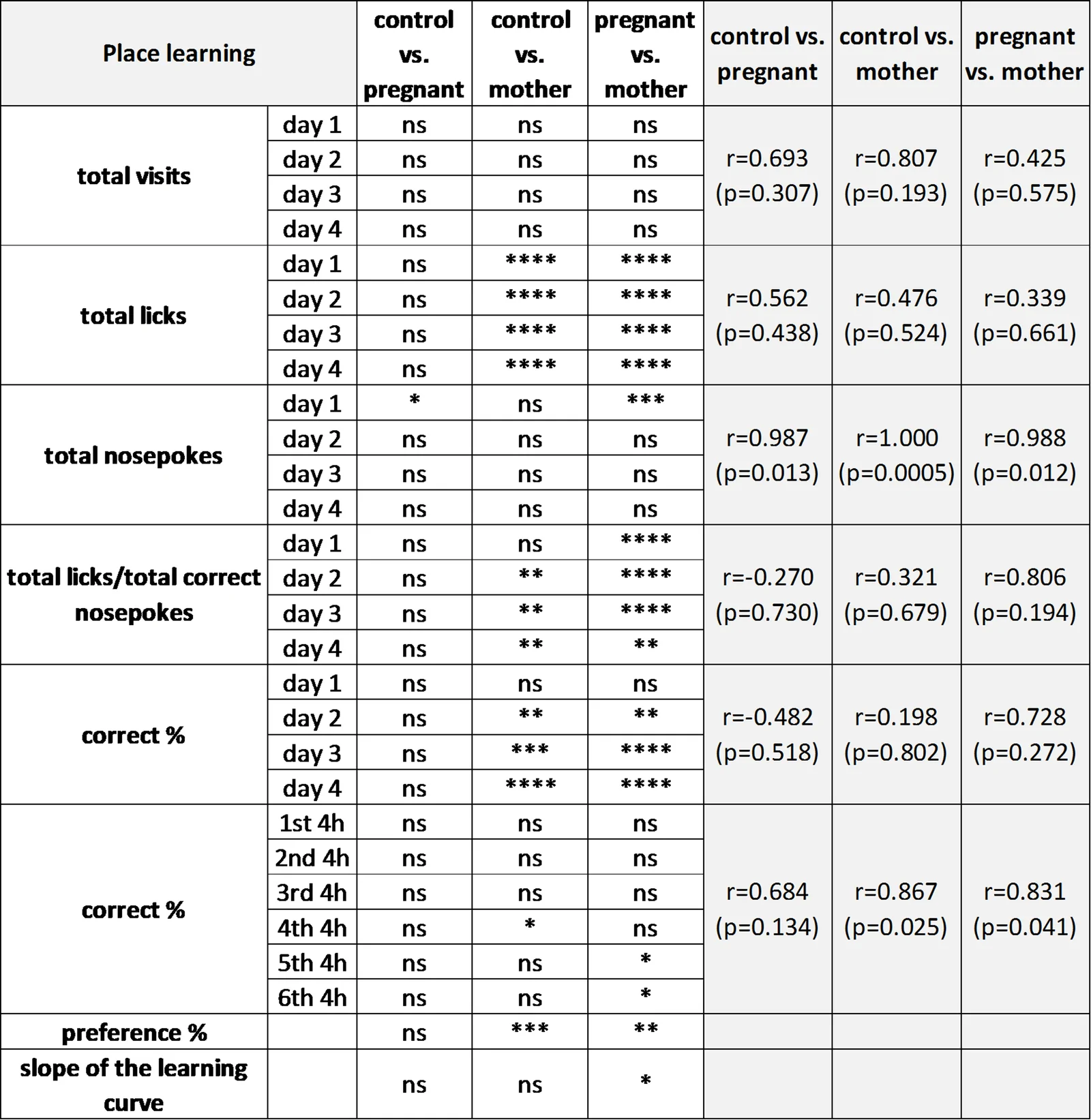
Place learning. Table shows the results of the statistical analysis. Pearson correlation coefficients (r) and their corresponding significance values (p) are shown in a grey background. *p < 0.05, **p < 0.01, ***p < 0.001, ****p < 0.0001

### Reversed place learning

**Fig. 5:**
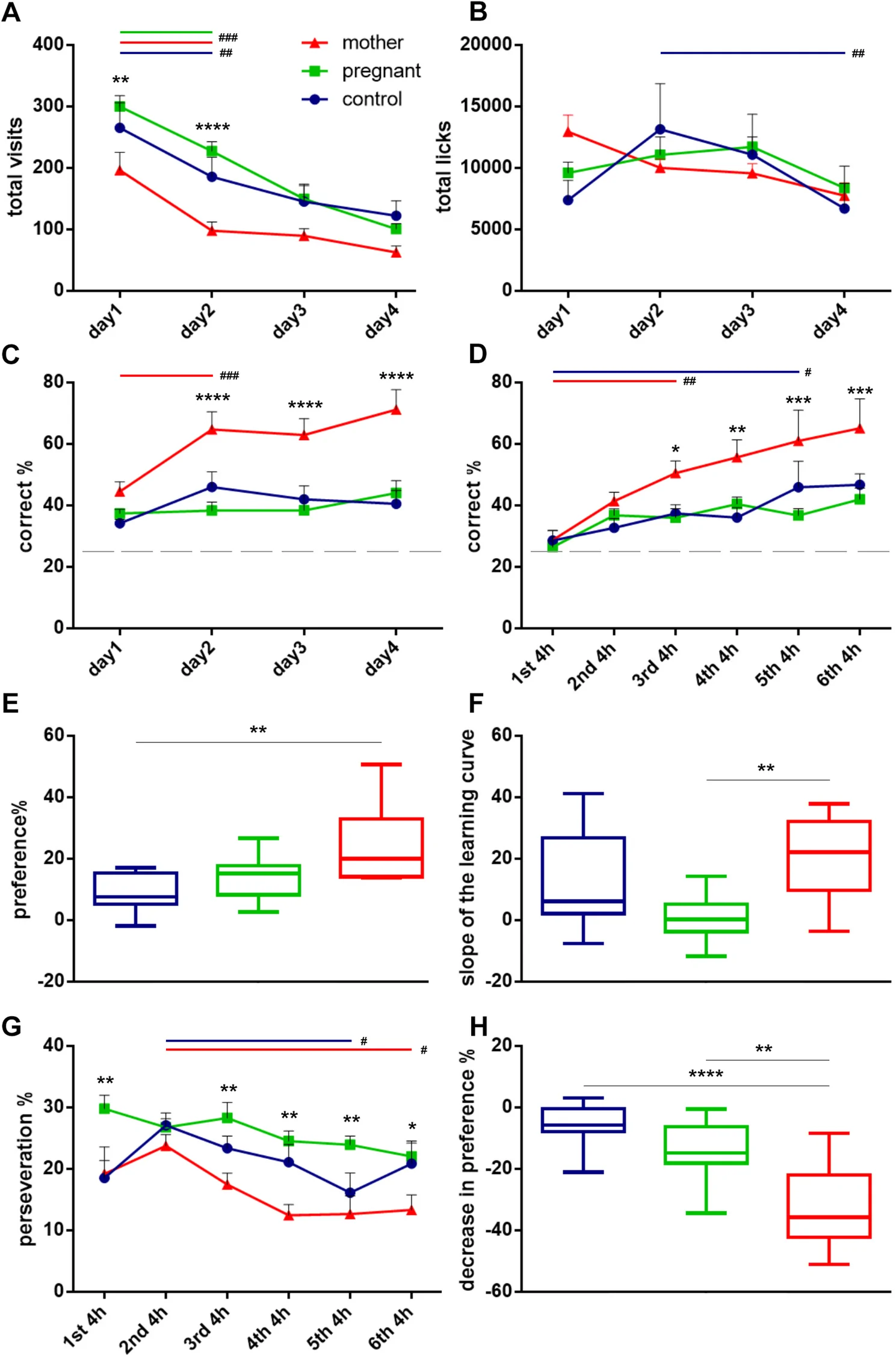
Reversed place learning phase. During the reversed place learning phase, animals could obtain water by entering and nose-poking the corner opposite to the one assigned during the initial place learning phase. **A** Significant differences in total visits were detected on day 1 and day 2 between pregnant (in mid-pregnancy) and mother (in late postpartum) mice. **B** No differences in the total number of licks over the 5 days were observed. **C** We found that mother mice made a higher percentage of visits to the correct corner from day 2 than control/pregnant mice. Additionally, correct% increased across the 5 days in all groups, illustrating learning. **D** We found that mother mice had a higher correct% than control/pregnant mice from the third 4 h of the first day. **E** There were also significant differences in preference % between control females and mothers. **F** The slope of the learning curve of the mothers between day 1 and day 2 also differed significantly from that of pregnants. **G** As the significantly reduced preference % indicates, on the first day of the reversed place learning, mothers exhibited diminished fidelity to the corner previously associated with the correct location during the initial place learning phase. **H **Compared to the control and pregnant mice, mother’s reduced preference for the previously correct corner—once it no longer yielded water. Data are presented as means +/-SEM. *p < 0.05, **p < 0.01, ***p < 0.001, ****p < 0.0001

Next, animals were assessed in the reversed place learning phase, where the correct corner was opposite to that of the first phase of place learning. We found significant differences in total visits, a decrease between day 1 and day 2 in all groups (Fig. 5A). The value in postpartum mothers changed similarly as in place preference. In the other 2 groups, a very high number of total visits were seen, particularly in the first 2 days of reversed place learning. Water consumption between control/mother mice and pregnant female mice was not significantly different (Fig. 5B). For correct%, we found a significant main effect of day (Fig. 5C), illustrating learning across the 4 days in postpartum mothers. In line with that, we also found that the mother mice had a significantly higher correct% from the second day compared to control and pregnant mice (Fig. 5C). We also examined the learning performance of the animals on the first day of this test at a resolution of 4 h, and found that the correct% was again significantly higher for the postpartum mothers compared to the control and pregnant females from the 3rd 4 h (Fig. 5D). In this case, preference% represents the difference of correct% between the last day of place learning and the first day of reversed place learning. There is a significant difference between control mice and postpartum mother mice with the latter group exhibiting higher correct% (Fig. 5E). Figure 5F shows the slope of the learning curve between day 1 and day 2, where a significant difference was found between the slope of the learning curve of mothers and pregnants. Perseveration in learning refers to the inability or difficulty in shifting from one task to another. We found that on the first day of reversed place learning, mothers were less adherent to the corner assigned as the correct corner in the place learning phase, which was reflected in their significantly lower perseveration % (Fig. 5G). Their preference for the corner previously designated as the correct corner was significantly reduced compared to the control/pregnant group when it no longer provided them with water, suggesting improved cognitive flexibility (Fig. 5H). The statistical data for the reversed place learning task are shown in Table 3.

**Table 3:**
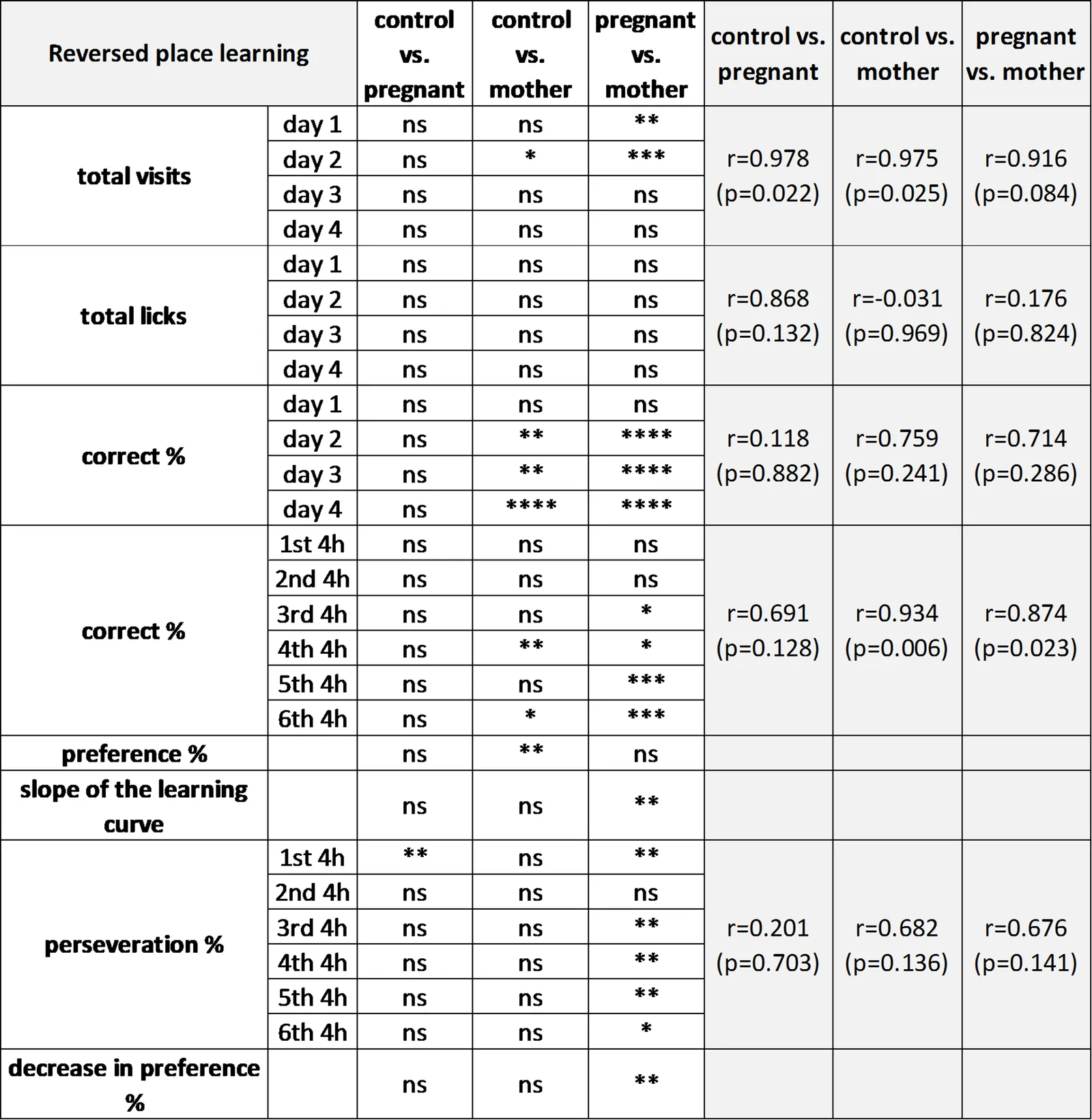
Reversed place learning. Table shows the results of the statistical analysis. Pearson correlation coefficients (r) and their corresponding significance values (p) are shown in a grey background. *p < 0.05, **p < 0.01, ***p < 0.001, ****p < 0.0001

### Fixed schedule drinking

**Fig. 6:**
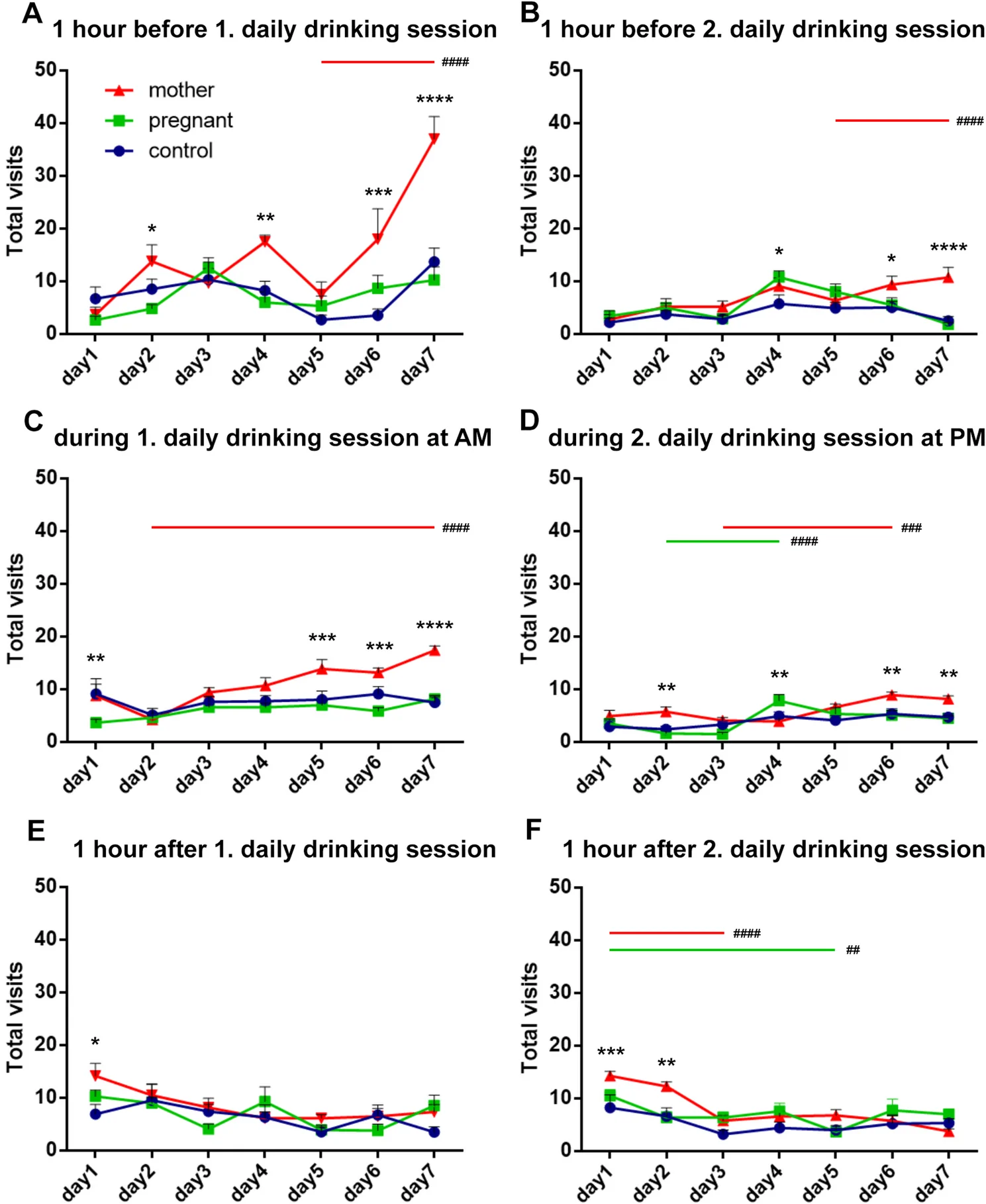
Total visits in fixed schedule drinking phase. Number of visits 1 h before water accessibility **A**,** B**, during water access **C**,** D** and 1 h after water accessibility **E**, **F**. Pregnant mice were in late pregnancy and mother mice were in early postpartum period for this task. Data are presented as means +/- SEM. *p < 0.05, **p < 0.01, ***p < 0.001, ****p < 0.0001

We focused on the one-hour periods before, during and after the access to water in order to analyze anticipatory and persistent behavior of mice. The number of visits increased in mother mice during the hours preceding water access as the days progressed, while for the other two groups, there was no similar change (Fig. 6A, B). This may be interpreted as an anticipation of the up-coming event (water access) and did not show persistent higher activity after water was not available anymore. The change was more significant during the first drinking session. During the hours when water was accessible, the number of visits showed significant differences between mother mice and control/pregnant (Fig. 6C, D). The number of visits during drinking sessions also showed a significant increase in mothers between day 2 and day 7 (during (1) drinking session) and between day 3 and day 6 (during (2) drinking session) (Fig. 6C, D). During the hours following water access, higher visits for mothers were observed in the first days as compared to control/pregnant mice, which diminished as the days progressed (Fig. 6E, F).

In the hour following the 2. drinking session, a significant effect of the days was found between day 1 and day 3 in the mothers (Fig. 6F). It is also important to note that we did not find any increase in the mortality of pups during the procedure. The statistical data for the fixed schedule drinking task can be found in Table 4.

**Table 4:**
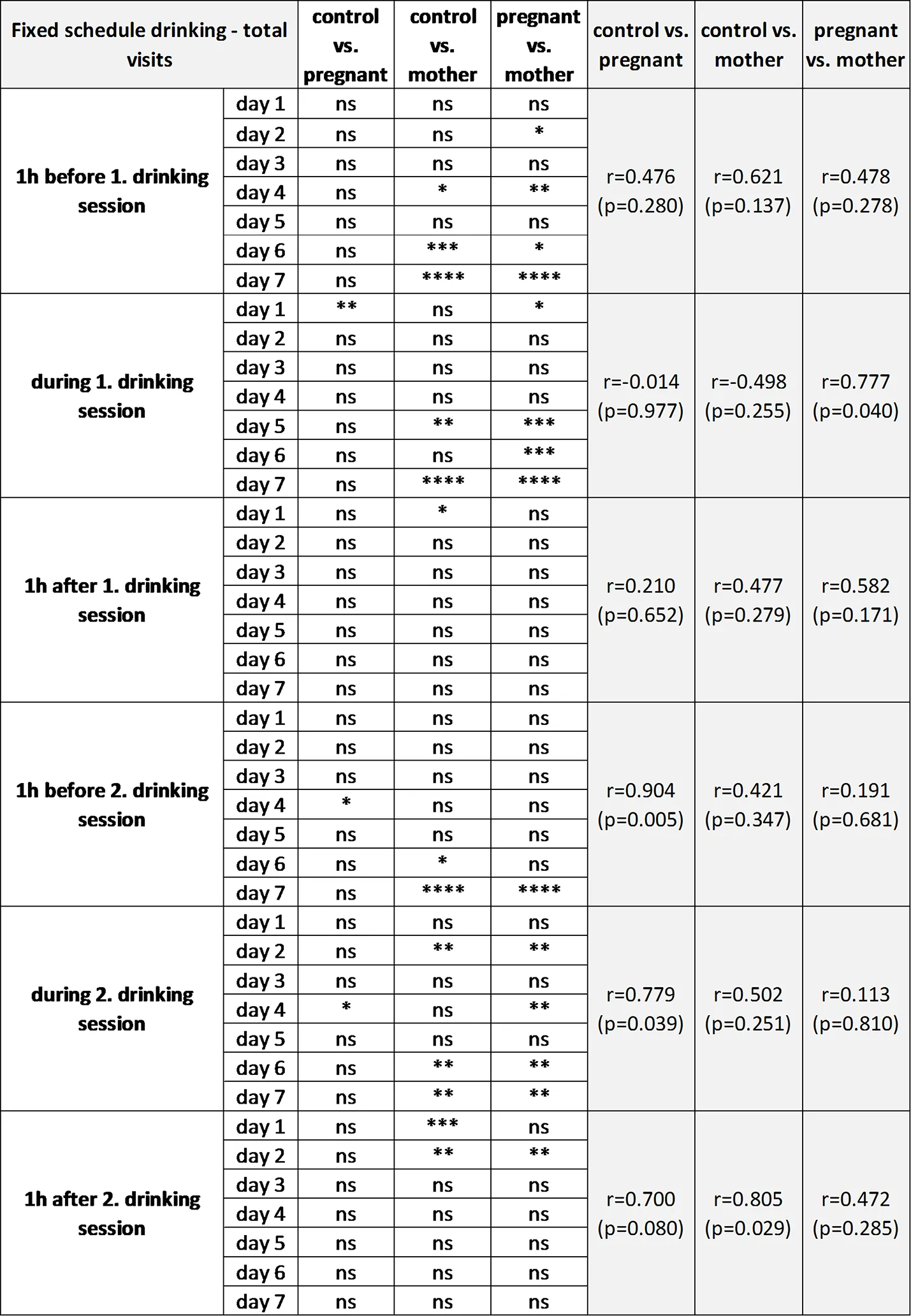
Total visits in fixed schedule drinking phase. Table shows the results of the statistical analysis. Pearson correlation coefficients (r) and their corresponding significance values (p) are shown in a grey background. *p < 0.05, **p < 0.01, ***p < 0.001, ****p < 0.0001

**Fig. 7:**
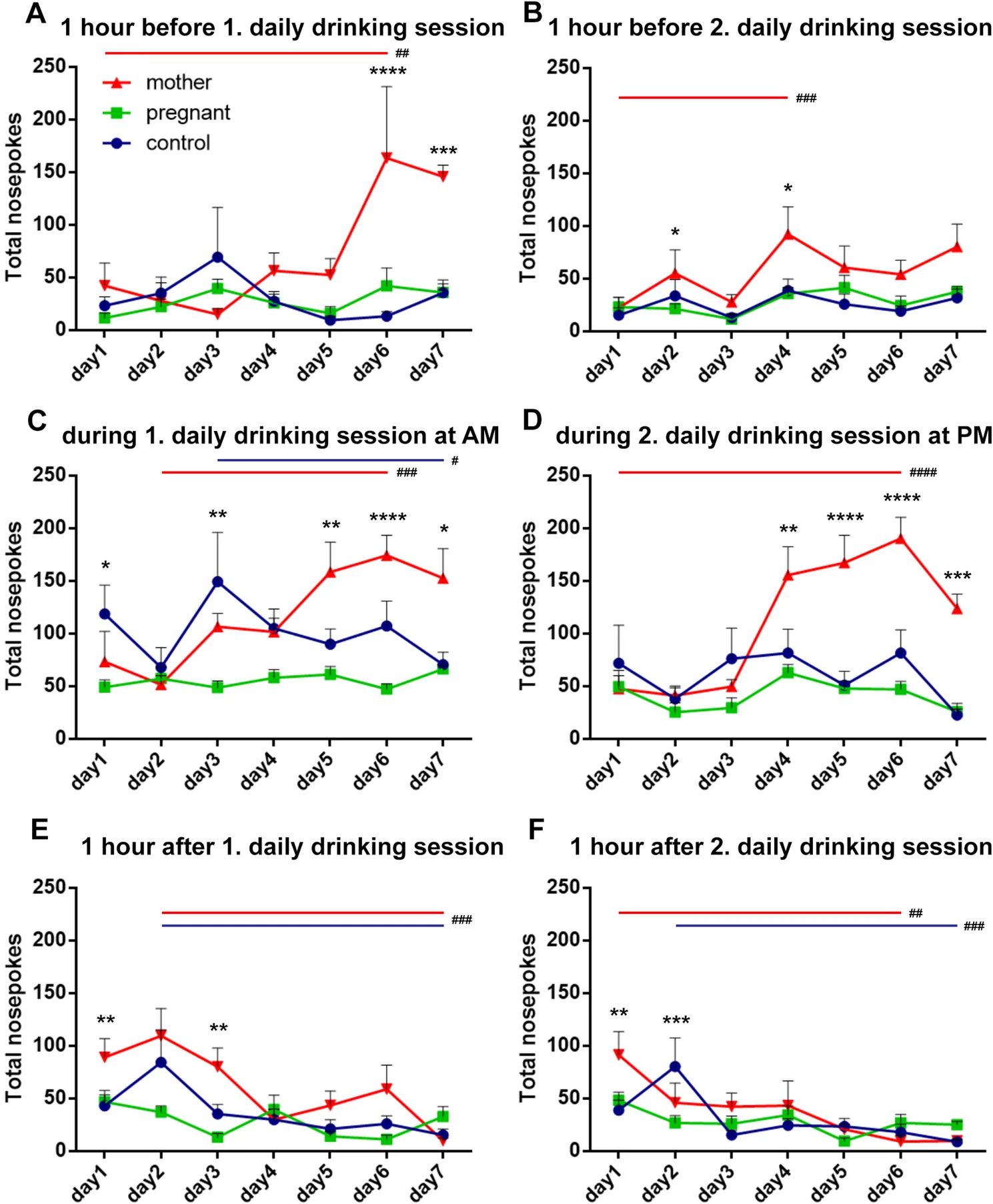
Total nosepokes in fixed schedule drinking phase. Number of nosepokes 1 h before water accessibility **A**, **B**, during water access **C**, **D** and 1 h after water accessibility **E**, **F**. Pregnant mice were in late pregnancy and mother mice were in early postpartum period for this task. *p < 0.05, **p < 0.01, ***p < 0.001, ****p < 0.0001

In the hours before drinking sessions, the number of nosepokes increased significantly in mothers as the days progressed (Fig. 7A, B). We also observed similar effect during drinking sessions (Fig. 7C, D). In the hours following drinking sessions, the number of nosepokes significantly decreased as the days progressed in both mothers and controls (Fig. 7E, F).

The number of nosepokes exhibited a proportional relationship with the total number of visits, resulting in curves that were similar to those observed for total visits in the hours before drinking sessions (Fig. 7A, B), during drinking sessions (Fig. 7C, D) and in the hours after drinking sessions (Fig. 7E, F). So not only the exploratory activity of the mothers increased during the hours preceding water access, but also the number of nosepokes specifically associated with drinking. The statistical data for the nosepokes in fixed schedule drinking phase are shown in Table 5.

**Table 5:**
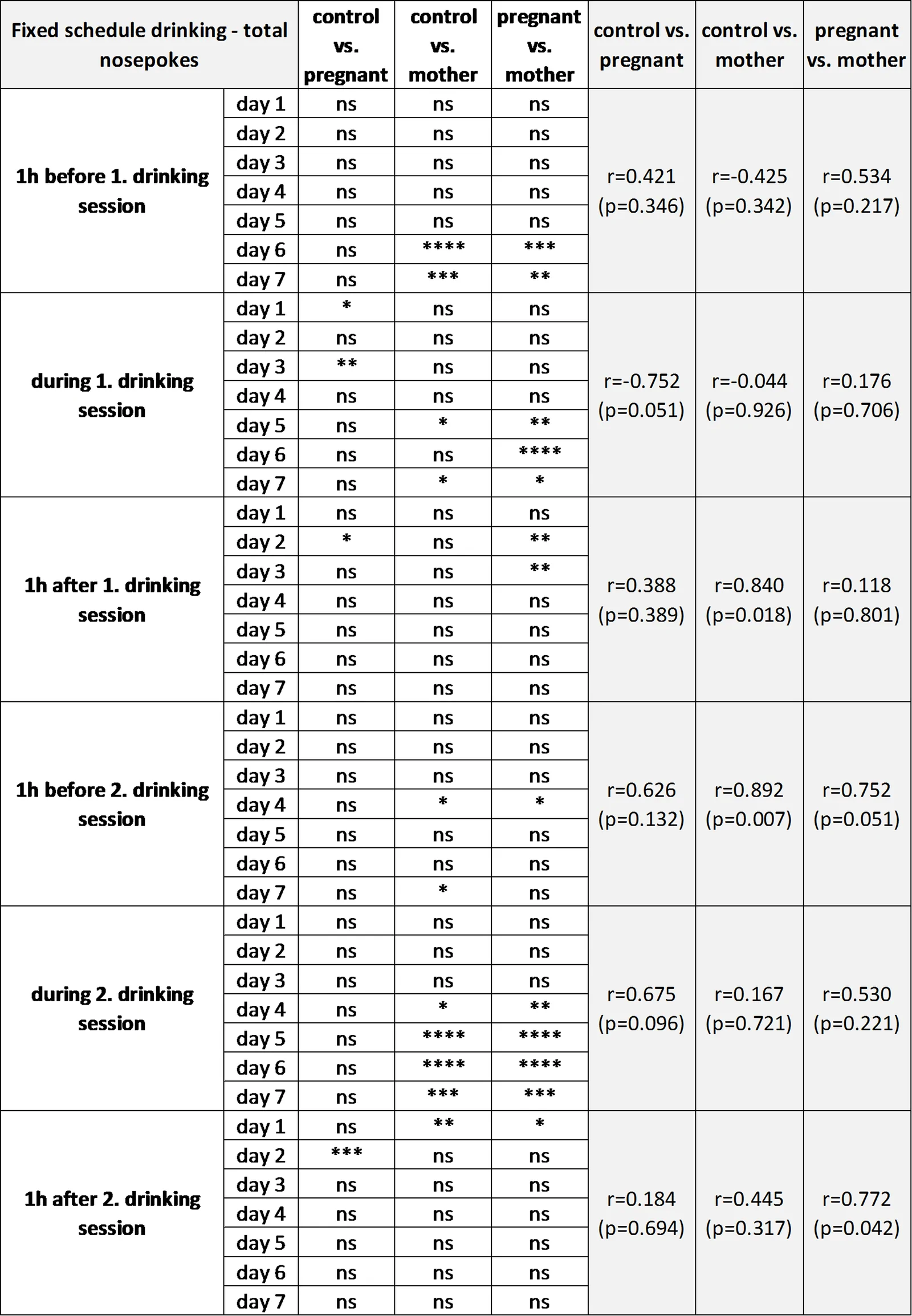
Total nosepokes in fixed schedule drinking phase. Table shows the results of the statistical analysis. Pearson correlation coefficients (r) and their corresponding significance values (p) are shown in a grey background. *p < 0.05, **p < 0.01, ***p < 0.001, ****p < 0.0001

**Fig. 8:**
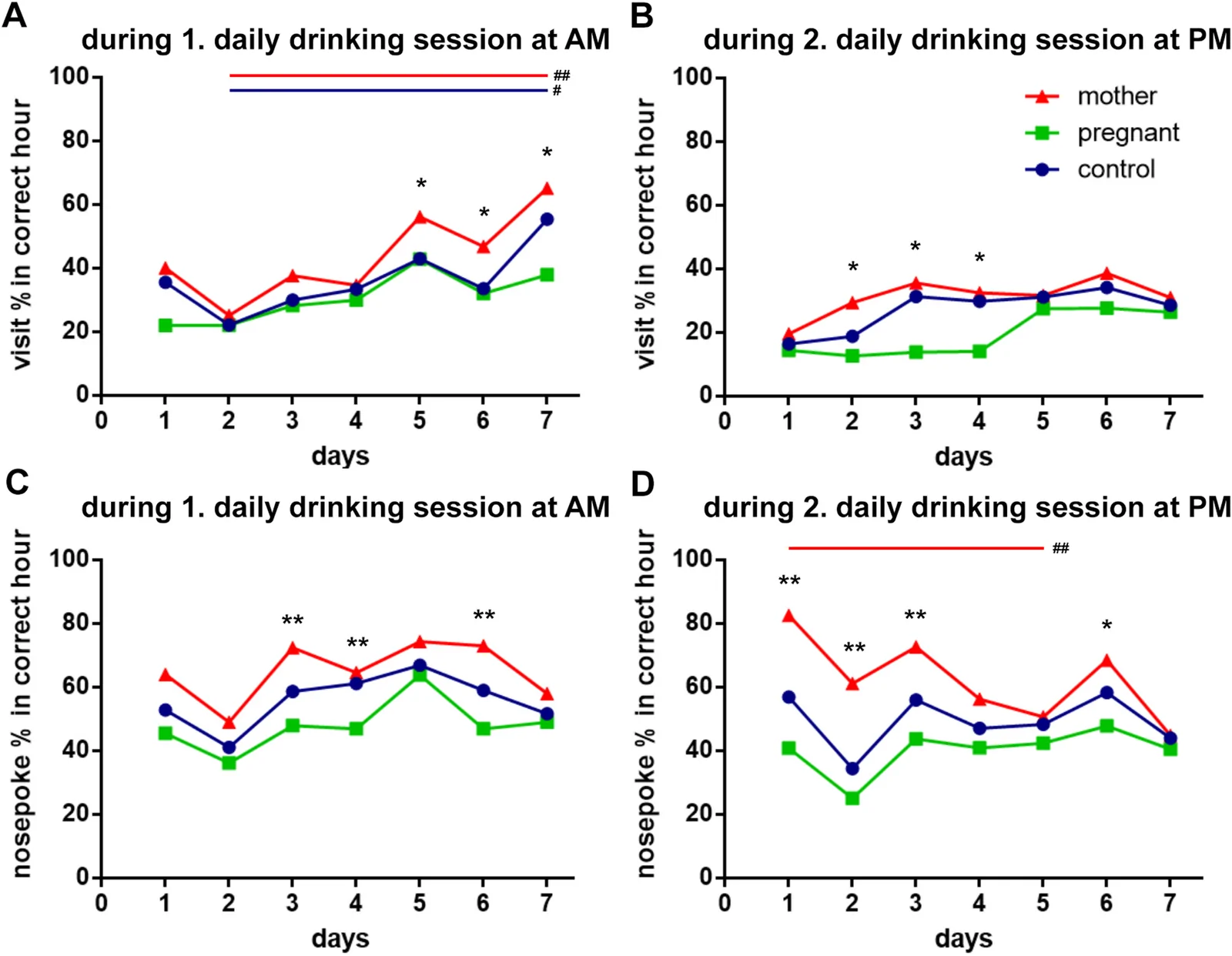
Visit% and nosepoke% in the correct hours of the fixed schedule drinking phase. The proportion of visits during water access compared to the total number of visits within the three-hour period (**A, B**). The proportion of nosepokes during water access compared to the total number of nosepokes within the three-hour period (**C, D**). *p < 0.05, **p < 0.01, ***p < 0.001, ****p < 0.0001

The ratio of visits at the (1) correct hours (AM) started to increase for mothers from day 4, as can be seen from the visit% in correct hour (proportion of visits occurred during the middle hour), with the (2) correct hours (PM) showing a difference between days 2 and 4 in favour of mothers (Fig. 8A, B). The number of nosepokes at both correct hours differs in favour of mothers on certain days (Fig. 8C, D). The statistical data for visit% and nosepoke% in the correct hours of the fixed schedule drinking phase are shown in Table 6.

**Table 6:**
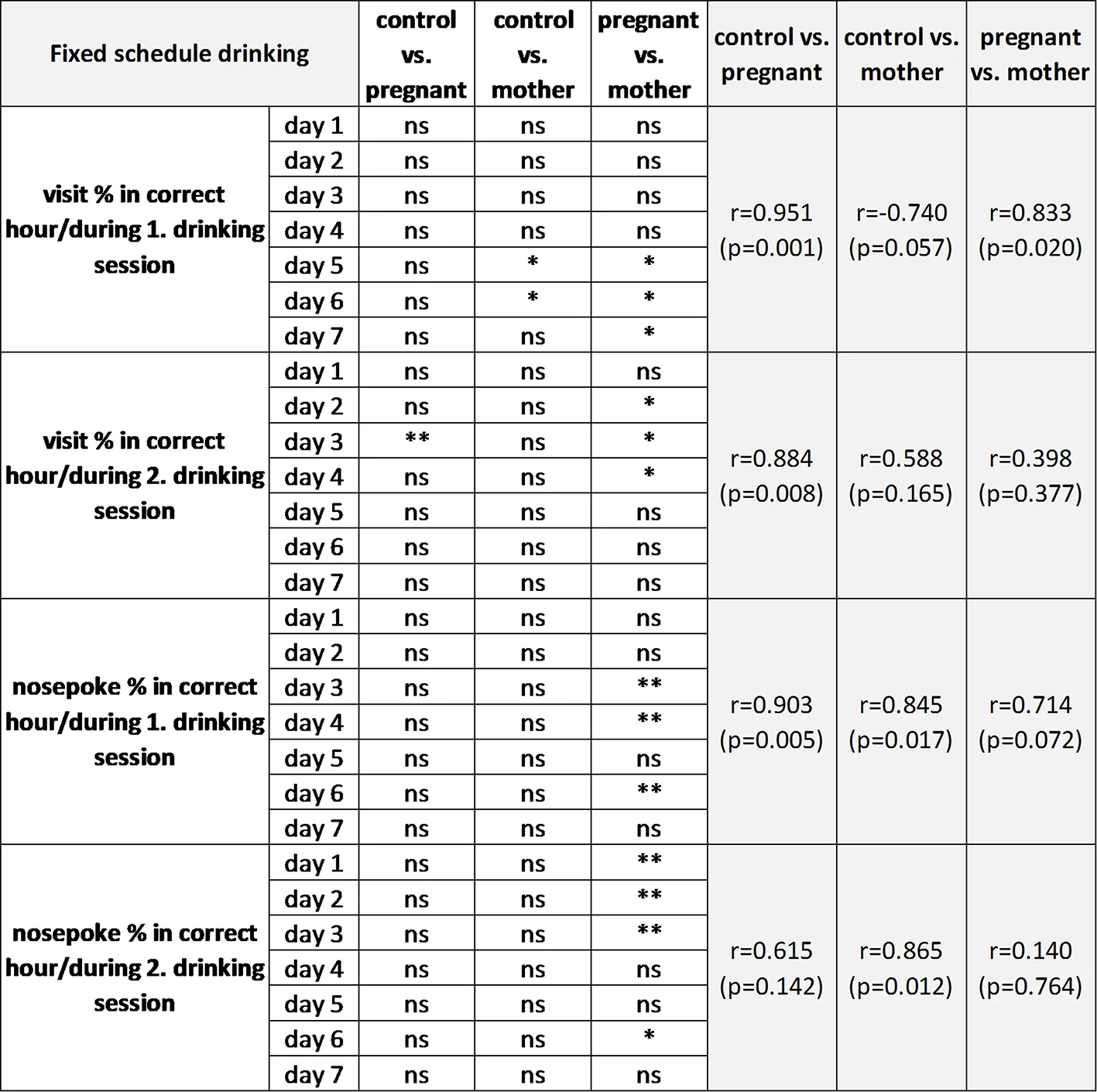
Visit% and nosepoke% in the correct hours of the fixed schedule drinking phase. Table shows the results of the statistical analysis. Pearson correlation coefficients (r) and their corresponding significance values (p) are shown in a grey background. *p < 0.05, **p < 0.01, ***p < 0.001, ****p < 0.0001

**Fig. 9:**
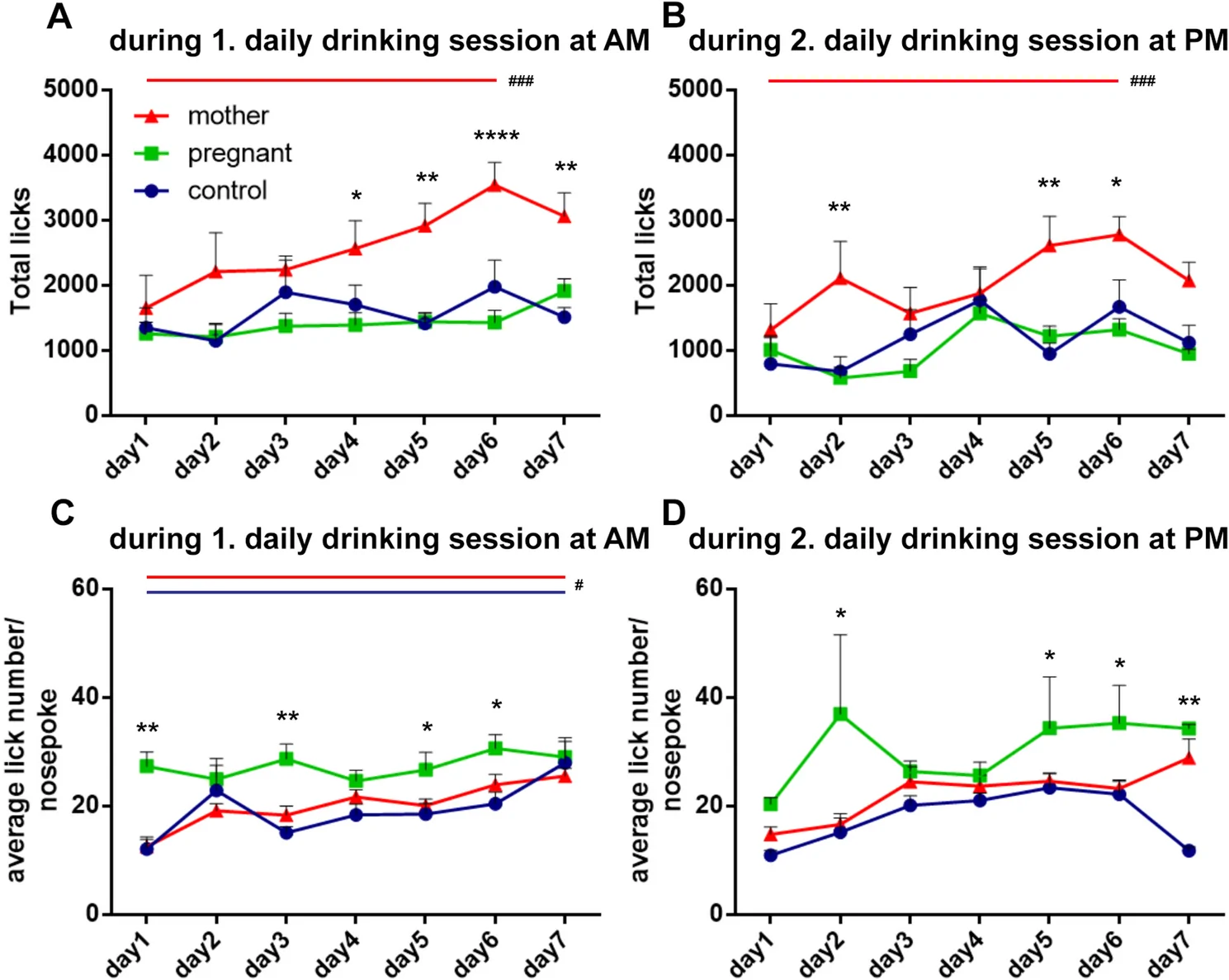
Drinking sessions in fixed schedule drinking phase. Number of licks during the first one-hour (from 10 AM) **A** and second one-hour (from 3 PM) **B** drinking sessions. Average lick number per nosepoke during the first one-hour (from 10 AM) **C** and second one-hour (from 3 PM) **D** drinking sessions. *p < 0.05, **p < 0.01, ***p < 0.001, ****p < 0.0001

During drinking session, there were significant differences in total licks between mothers and control/pregnant mice (Fig. 9A, B). The average lick number per nosepoke also differs significantly between groups, but in this case in favour of pregnant mice (Fig. 9C, D).

In drinking sessions, not only the number of nosepokes but also the number of licks showed a significant increase with the progression of days in mothers (Fig. 9A, B). Days had a significant effect on the number of licks per nosepoke in the first drinking session in mothers and control mice (Fig. 9C, D). The statistical data for drinking sessions in fixed schedule drinking phase are shown in Table 7.

**Table 7:**
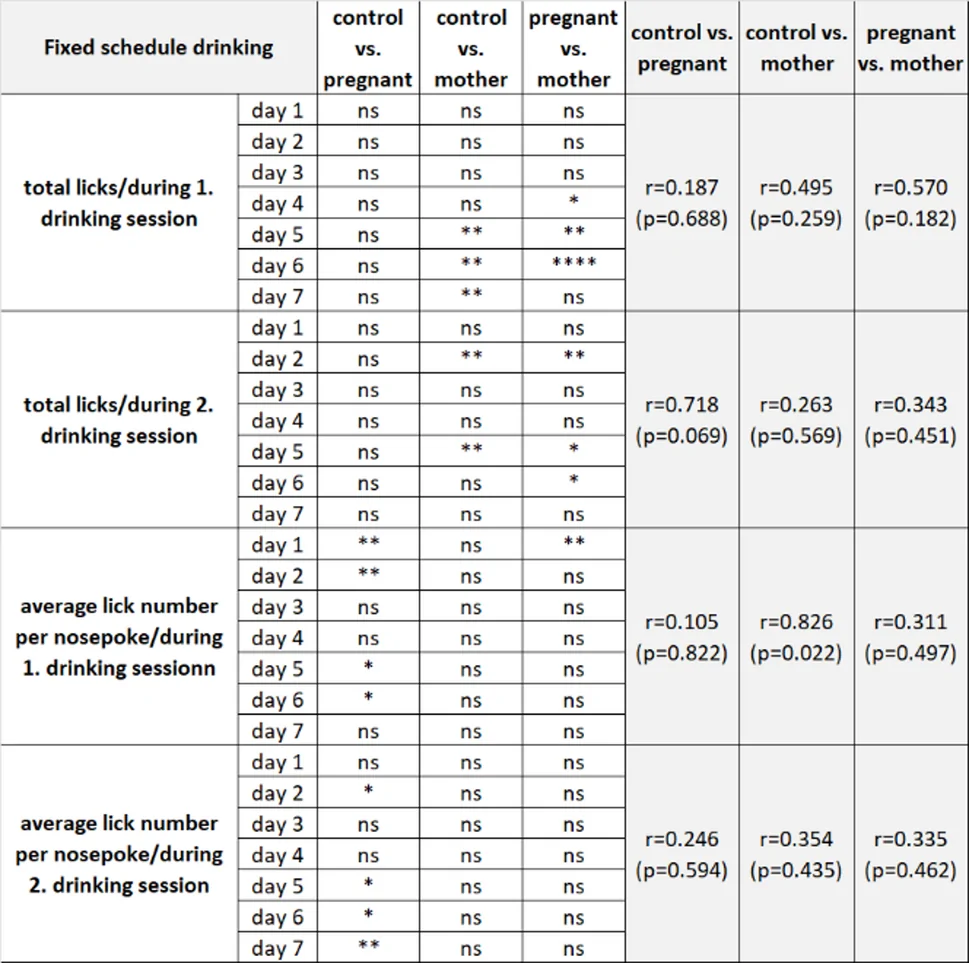
Drinking sessions in fixed schedule drinking phase. Table shows the results of the statistical analysis. Pearson correlation coefficients (r) and their corresponding significance values (p) are shown in a grey background. *p < 0.05, **p < 0.01, ***p < 0.001, ****p < 0.0001

**Fig. 10:**
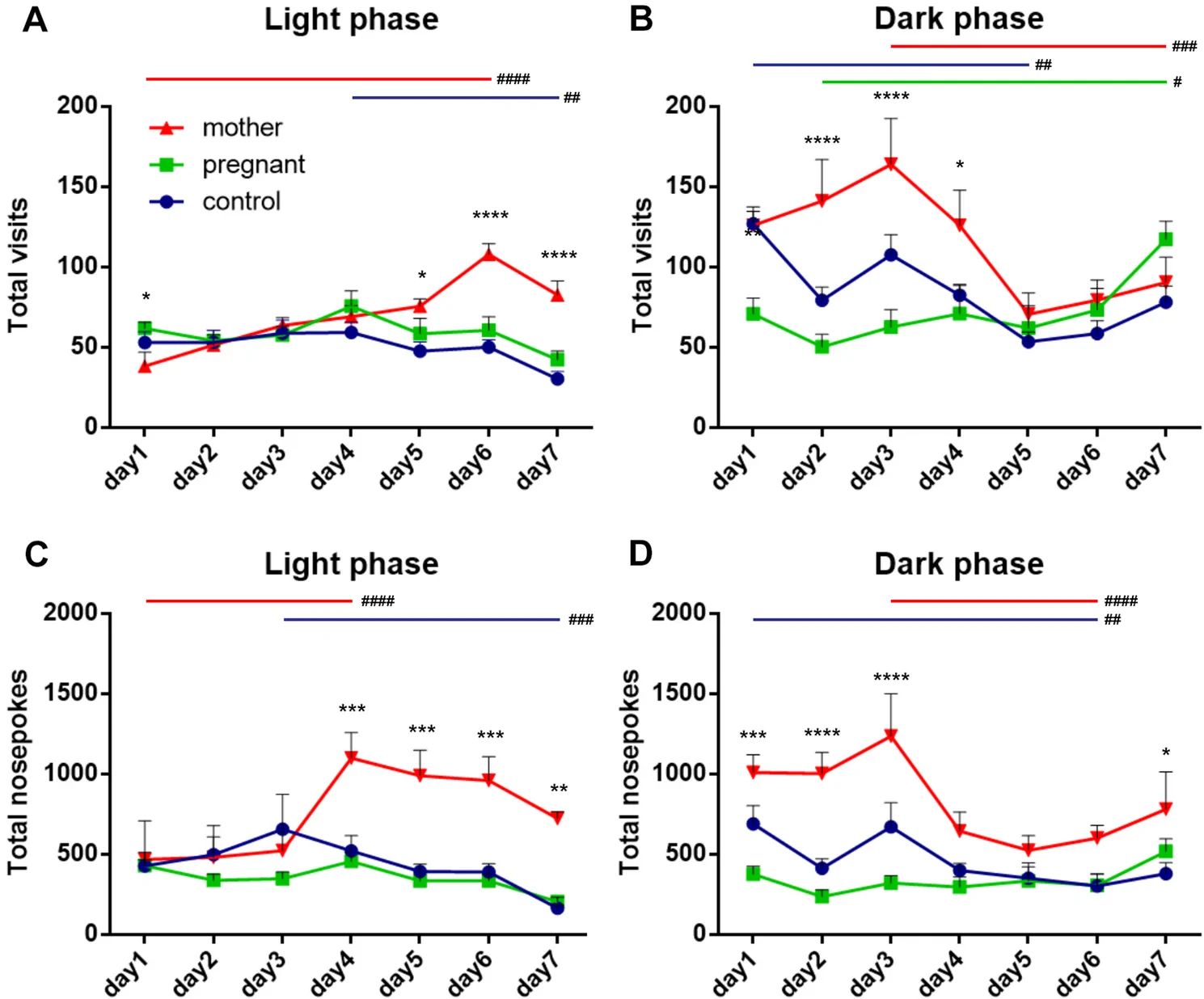
Number of visits and nosepokes in 12-hour bins during fixed schedule drinking. Total number of visits during light phase (**A**). Total number of visits during dark phase (**B**). Total number of nosepokes during light phase (**C**). Total number of nosepokes during dark phase (**D**). *p < 0.05, **p < 0.01, ***p < 0.001, ****p < 0.0001

Mother mice modified their corner visit activity after 4 days, as the difference between dark and light periods reversed **(**Fig. 10A, B**)**, with significant changes in the number of nose-pokes as well (Fig. 10C, D). Mother mice decreased their number of corner visits during the dark phase and increased in the light phase. However, a marked difference remained between dark and light periods. The same happened with nosepokes around day 4, with the previous higher number of nosepokes during dark phase being replaced by a higher number of nosepokes during light phase. Meanwhile, the number of visits and nosepokes of control/pregnant females did not change significantly during the 7 days, so they were not able to change their behavior according to the changed conditions. Overall, control/pregnant mice displayed a significantly lower number of visits and nosepokes during the light periods from day 4 and no increase in these parameters was observed later on.

The number of visits in light phase showed a significant increase in mothers and a significant decrease in controls over the days, while in the dark phase there was a decrease in the number of visits in mothers and control animals and a significant increase in pregnants (Fig. 10A, B). A similar pattern was observed in the number of nosepokes with the progression of days (Fig. 10C, D). The statistical data for the number of visits and nosepokes in 12-hour bins during fixed schedule drinking are shown in Table 8.

**Table 8:**
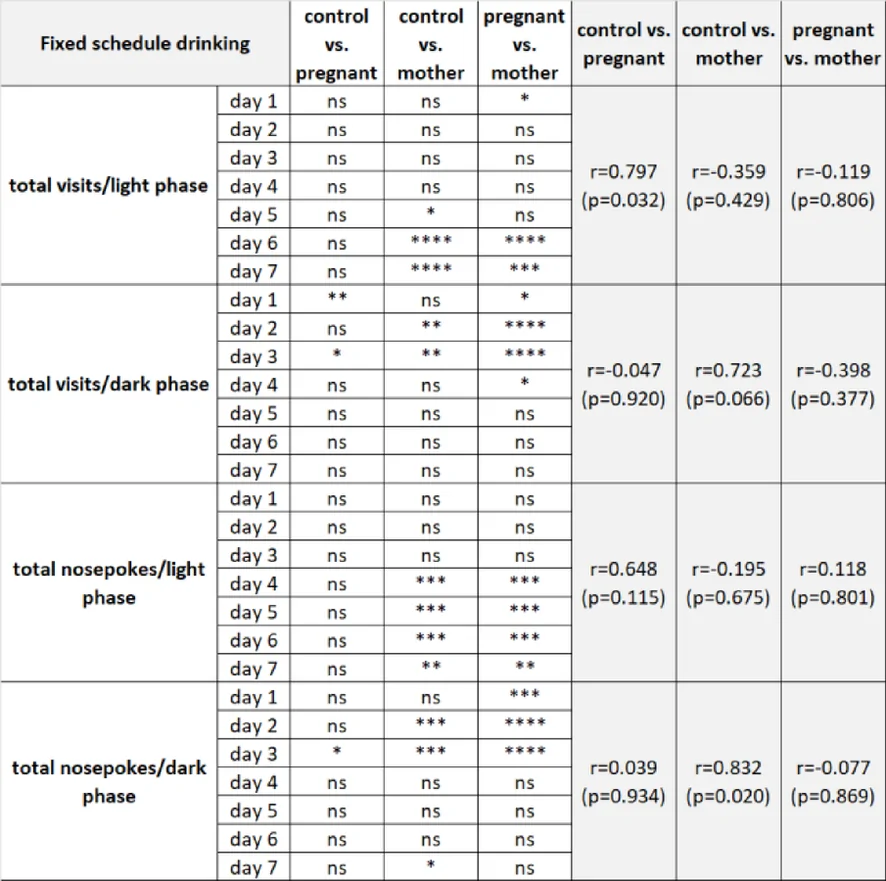
Number of visits and nosepokes in 12-hour bins during fixed schedule drinking. Table shows the results of the statistical analysis. Pearson correlation coefficients (r) and their corresponding significance values (p) are shown in a grey background. *p < 0.05, **p < 0.01, ***p < 0.001, ****p < 0.0001

## Discussion

The present study is the first to determine that motherhood improves spatial learning performance and cognitive flexibility using IntelliCage. Previously, standard behavioral tests were employed to examine these phenomena in relation to different reproductive stages, but the results were limited [2, 17, 43]. In turn, the present results revealed significant enhancements in spatial learning and cognitive flexibility in postpartum female mice compared to pregnant and control animals. It is important to note that the composition of the three experimental cycles differed and could change if animals gave birth during the experiment. Thus, in the first cycle, it was possible that the animals were placed in the pregnant group until the start of the fixed-schedule drinking task. After giving birth, the same animals were placed in the mothers’ group at the start of the fixed-schedule drinking task. In the second cycle, the animals began the adaptation period in the IntelliCage as mothers. In the third cycle, the animals gave birth after the experiments finished, so they performed all learning tasks as pregnant animals. This complicates the interpretation of the results. There could have been something different in the second cycle (e.g., the experimenter, outside noises, humidity, etc.), or the different group composition and interactions could have contributed to the results. Despite these considerations, we believe the experiments are largely comparable because all other experimental parameters were identical across groups. Any observed differences in learning and behavioral flexibility are likely due to reproductive status rather than other procedural variables.

### The effect of the reproductive status on different types of learning

During the four-day adaptation period, all animals exhibited signs of acclimatisation to the IntelliCage, as evidenced by frequent visits to all corners and the acquisition of corner-drinking skills through nosepoking. During this process, the initially high visit and nosepoke numbers declined from day 1 to day 2 in all groups and remained stable thereafter. This decline can be interpreted as a result of the elevated exploration observed on the first day of habitation in the IntelliCage, during which the animals familiarised themselves with the corners and nosepoking devices. Concurrently, the total lick numbers exhibited relative stability, attributable to the increase in visits with lick/total visit and the lick number/nosepoke ratios on the second day. This suggests that by that day, the animals had acquired the ability to utilise the nosepoke for obtaining a drink to lick. While these tendencies were evident across all groups, variations in behavior were observed among different groups. A notable observation was the increased exploratory drive exhibited by pregnant mice, as evidenced by their frequent visits to corners, in contrast to the behavior patterns of the other groups. While no other study has been found that investigates the altered exploratory drive of pregnant animals, the finding of increased exploration could be considered as a preparation for finding a place to build a nest. However, it is important to note that anxiety can also reduce exploratory behavior. Therefore, reduced anxiety in pregnant animals could also explain the initially increased exploratory behavior in the pregnant group. A further significant disparity between the groups was observed in the elevated licking behavior exhibited by the postpartum mothers. Given the constant size of the liquid drop in the IntelliCage, the elevated licking number is indicative of increased fluid intake, a phenomenon that is well supported by the literature and is entirely consistent with the needs of a postpartum animal that is responsible for nourishing her offspring. However, this makes the value of the fluid reward higher in the postpartum group than in the other two groups, implying that the reward value differs among groups and making it difficult to equate learning and performance. It is also important to note that while food is freely available for the animals in the IntelliCage, the amount of water and food intake is proportional due to the strict osmotic regulatory processes if the salt content of the food is constant [23], as was the case in our experiment. The increased lick number in mothers was primarily due to an elevated lick number per nosepoke within the 5 s during which licking was possible following a nosepoke. However, the number of total nosepokes was also somewhat higher in mothers than in the other groups. This finding is significant because the number of nosepokes is proportional to the motivation of the appetitive phase of the feeding behavior (food seeking). In contrast, the intensity of licking may be associated with the consummatory, hedonic motivation. These two phases of feeding behaviors are potentially governed by distinct brain mechanisms [22]. On the first day of the place learning, the animals were suddenly unable to access water in three of the four corners. This led to an increase in the number of visits made by the animals, particularly by the mothers, which decreased by the third day, with the mothers visiting the corners less frequently than the other groups. This decline in exploratory behavior among mothers is consistent with previous findings in rats [18].

In place learning, the animals’ acquisition of the correct corner for drinking was elevated. The ratio of visits to this corner increased twofold in control and pregnant animals, and threefold in mothers during the first 1.5 days. The initial ratio of visits to the correct corners was slightly less than the 25% by chance ratio because the correct corner for each animal was selected to be an originally less preferred one to avoid preference bias. While all groups demonstrated learning of the correct corner, a pronounced difference emerged among the groups. Mothers exhibited a significantly faster rate of improvement in the correct corner ratio compared to the other groups. This finding is further supported by the observation that the difference in correct% measured between the final day of nosepoke adaptation and the initial day of place learning (termed Preference%) was significantly higher in the mother group than in the other groups. Additionally, the mother group exhibited a steeper correct% curve on the first day of place learning. Furthermore, the ratio of visits to the correct corner continued to improve beyond the first day. Consequently, during the process of place learning, postpartum females exhibited a more rapid acquisition of the correct location associated with water and a more pronounced improvement in performance over time. The elevated lick number observed in mother as compared to the other groups despite a concomitant reduction in total visitation by mothers can be attributed to increased nosepoking or increased licking/nosepoke ratio. These two possibilities represent big differences in interpretation as well as in brain mechanisms because in operant paradigms, appetitive (seeking) behavior is typically quantified by instrumental responding such as lever pressing or nosepoking, whereas consummatory (taking) behavior, which is likely controlled by different mechanisms, is measured via licking or intake-related parameters [7, 15, 35, 42]. Since we could not find previous literature on the separate assessment of appetitive and consummatory behavior, we used total nosepokes as an indicator of appetitive motivation and the average number of licks/correct nosepokes as an indicator of consummatory behavior, based on general behavioral literature. For the place learning phase, we concluded that only consummatory, not appetitive, motivation for drinking was elevated. This suggests that improved place learning was not a consequence of increased motivation to drink, but rather an increased cognitive ability of the mothers. This is also supported by the finding that the non-mother groups never reached the same percentage as the mothers, which rules out the possibility that only learning speed was affected. The results of improved place learning in mothers are consistent with previous data obtained in the rat, which demonstrated that maternal rats outperform virgin females in spatial tasks [38]. Maternal rats demonstrate a reduced number of errors and exhibit enhanced efficiency in navigating the Morris water maze and object placement tasks [46]. It is noteworthy that these cognitive enhancements are specific to spatial learning, as maternal and virgin rats demonstrate equivalent performance in object recognition [27]. In a similar vein, during the phase of reversal learning, the temporal progression of the decline in total visits and the rise in correct corner ratio, along with other attributes of the adaptation learning, exhibited parallels with those found in place learning. It was also observed that postpartum females demonstrated greater adaptability to the new reward location. The enhanced outcomes in reversed place learning in IntelliCage are generally interpreted as indicative of augmented cognitive flexibility, given its association with the capacity to adapt behavior in response to altered rules or contingencies, employ alternative strategies when prior ones are no longer effective, and suppress established responses in favour of new ones [9, 30]. Perseveration in learning refers to the inability or difficulty in shifting from one task to another. On the first day of the reversed place learning phase, the mothers were less likely to adhere to the corner that had been designated as correct during the initial place learning phase. This is reflected by their significantly lower perseveration percentage. Compared to the control/pregnant group, their reduced preference for the corner that no longer provided water reinforcement suggests enhanced cognitive flexibility and a greater ability to update previously learned associations. This finding suggests that maternal experience can facilitate adaptive behavioral responses when task contingencies change. Such improvements in cognitive flexibility may be linked to neurobiological adaptations associated with motherhood. These adaptations include alterations in prefrontal cortical plasticity, dopaminergic signaling, and stress reactivity, which support efficient learning and behavioral adjustment [6, 40]. Therefore, it can be concluded from the findings that mother mice possess superior cognitive flexibility in comparison to both controls and pregnant animals. It is also noteworthy that enhanced spatial learning and cognitive flexibility may confer significant survival advantages by enabling mothers to navigate their environment efficiently, secure resources, and manage the challenges of caregiving.

In the fixed schedule drinking paradigm, mice were provided with water at two-hour intervals, thereby reducing the degree of water restriction and enabling the collection of data twice daily. The distribution of these two hours was not uniform throughout the day; rather, they were separated by a period of five hours (or 19 h). Consequently, both drinking sessions occurred during the light phase, thereby prompting varying degrees of motivation to drink, owing to the heightened water deprivation following the extended 19-hour interval between sessions. However, the total licks and the number of licks following a nosepoke were not different between the two drinking sessions. In both cases, an analysis of day-to-day alterations revealed a significant increase in the total licks of the mothers as the schedule progressed, while the other two groups only exhibited a tendency to do so. Of particular interest is the finding that this increase in the total licks of mothers was largely due to an elevation of nosepoking activity between days 3–5, while only a small gradual increase in the number of licks per nosepoke was found in mothers suggesting increased appetitive motivation in mothers. In contrast, the latter parameter exhibited elevated levels in the pregnant groups from the outset, with the values of the mothers and controls only attaining parity by the seventh day of the schedule. This suggests that the elevated levels of licking per nosepoke observed in mothers remained constant during the fixed schedule of drinking in mothers, yet it increased similarly or even more than in the Adaptation phase in pregnant and control animals.

In addition to the drinking sessions, the number of visits and nosepokes was analysed during the hour when the animals had access to water, as well as during the hour before and after this, in order to ascertain whether mice are capable of learning time-based contingencies. It was observed that only the mother mice exhibited an increase in visiting and nosepoking activities prior to the drinking sessions. The elevated exploration levels were observed to commence on day 4 and to increase until day 6. This anticipatory parameter is used to assess temporal learning, and it can be concluded that postpartum mothers have a superior cognitive capacity for time tracking in comparison to controls and pregnant subjects. Alternatively, the elevated anticipatory behavior may be attributable to increased motivation, given that both changes in capability and motivation have the capacity to affect behavior [13].

A marked difference in behavior was also observed between the groups following the drinking session. Mother mice, and to a lesser extent the control group, persisted in their visits and nosepoking, while the pregnant subjects did not. This behavior was no longer observed by the third day of the fixed drinking schedule, indicating an effective extinction learning process. An examination of visit and nosepoke activities in periods outside of drinking sessions revealed a significant group difference, too. In the dark phase (when no drinking session was scheduled), mothers exhibited a significantly higher number of visits and nosepokes compared to the control and pregnant animals. A marginal increase in these behaviors was observed in the control group when compared to the pregnant group. These behaviors diminished between days 3 and 5, suggesting extinction learning. Conversely, a marked increase in visit and nosepoke behavior was observed in mothers during the dark phase (D3-D5), indicating a potential learning process related to the established schedule. In contrast, a decline in visits and nosepokes was observed in the light phase, a phenomenon that was particularly evident in the pregnant and control groups.

The increase in the number of visits during the correct hours observed in mothers from day 4 onward suggests that they learned the timing of the task more efficiently than the control or pregnant groups. Their enhanced performance during the correct hours, reflected by the higher number of nosepokes, indicates an improved ability to anticipate reward availability based on time cues. This performance may reflect an improved integration of temporal and spatial information, suggesting enhanced cognitive flexibility and learning efficiency. The temporal learning advantage observed in mothers may be related to neurobiological adaptations associated with motherhood that support more efficient goal-directed behavior. Thus, fixed schedule drinking tasks highlighted increased anticipatory and persistent behaviors in postpartum females, reflecting heightened motivation. These findings suggest that the postpartum period is associated with enhancements in multiple cognitive domains. Nevertheless, we cannot rule out the possibility that subtle social or olfactory cues between cage mates may have influenced the development of time-related learning. Future studies could clarify the potential contribution of observational learning in fixed schedule tasks.

By leveraging the IntelliCage system to study these adaptations, this research contributes to a growing body of literature on the interplay between reproduction and cognition. It underscores the importance of considering maternal status as a key variable in studies of cognition and behavior. Furthermore, these findings have broader implications for understanding the neurobiological basis of cognitive flexibility and its potential modulation by hormonal and environmental factors. Future research could explore the molecular and cellular mechanisms underlying these cognitive changes and their relevance to human maternal behavior and clinical conditions characterized by cognitive impairments.

### Potential mechanisms how the reproductive status can affect cognition and motivation

Spatial learning and cognitive flexibility are complex cognitive functions regulated by interconnected neural circuits, including the hippocampus, and the prefrontal cortex. The hippocampus, in particular, is critical for spatial navigation and memory [14], while the prefrontal cortex supports executive functions such as cognitive flexibility and decision-making [26]. Both regions undergo significant plasticity during pregnancy and lactation, as evidenced by changes in dendritic structure, synaptic connectivity, and neurogenesis [41]. However, the available evidence of neuronal plasticity in the postpartum period is more extensive in the hippocampus. Tomizawa et al., found that multiparous mice showed enhanced and sustained long-term potentiation (LTP) during the early postpartum period when compared to nulliparous mice [43]. Cell proliferation and dendritic complexity are reduced in the hippocampus of maternal rats during the early postpartum phase, in contrast to virgin females [10]. Additionally, postpartum neurogenesis has been linked to enhanced learning and memory in mothers. During weaning, when hippocampal neurogenesis is suppressed in rat dams [38], spatial learning and memory show improvement [38]. Lactation itself may influence hippocampal neurogenesis, as the effects of pregnancy and birth on neurogenesis do not persist in the weeks following weaning [33]. Increasing nursing demand in rats led to reduced hippocampal neurogenesis, as evidenced by fewer immature neurons and decreased cell proliferation at weaning [11]. In addition to lactation, other factors may influence hippocampal neurogenesis, such as interaction with offspring. Nulliparous sensitized female rats have more neurogenesis in the hippocampus, both via cell proliferation and new cell survival, compared to either postpartum rats or nulliparous female rats not exposed to pups [36].

It is hypothesised that the alterations in neuroplasticity that occur during the postpartum period are attributable to the significant hormonal fluctuations experienced during pregnancy, labour, and the postpartum phase. The ensuing neuroplasticity may serve as the basis for the enhanced cognitive abilities observed in postpartum females. The postpartum-specific alterations in neuroplasticity are hypothesised to be driven by hormonal changes that are characteristic of this reproductive stage [3]. Steroid and peptide hormones undergo significant fluctuations during the course of pregnancy, parturition and the postpartum period. Amongst these hormones, oxytocin is a potential mediator of enhanced hippocampus-dependent spatial learning in mothers. In a study by Tomizawa et al., oxytocin injections were found to enhance reference memory in nulliparous mice, yet no improvement was observed in working memory [43]. Oxytocin, has also been suggested to play a role in improving cognitive flexibility because oxytocin receptor antagonism in the medial prefrontal cortex blocked the enhanced cognitive flexibility observed in mother rats [28]. Additionally, ovarian hormones may also be involved, as high endogenous levels of estradiol (during proestrus) have been linked to impaired spatial learning, while low levels of estradiol have been associated with facilitated spatial learning [20]. Estradiol levels increase as the pregnancy progresses. By late pregnancy, estradiol levels rise in both humans and rodents, and high estradiol is associated with worsened spatial abilities in female rats [39]. However, following parturition, estradiol levels decline and remain low during the lactational anoestrus period. This reduction in estradiol has been linked to enhanced performance in female rats [19]. With regard to the performance of working memory in mother mice, corticosterone may play a significant role. It has been demonstrated that females that have not been pregnant or given birth exhibit enhanced working memory performance when exposed to chronic stress, which is associated with elevated corticosterone levels [32]. Given that basal corticosterone levels are elevated during lactation, it seems reasonable to posit that corticosterone plays an active role in spatial learning and memory enhancement. Another hormone with an elevated level in the postpartum period is prolactin. Surprisingly, the roles of prolactin, which is important for lactation and maternal behaviors, as well as adrenal hormones, in memory linked to the hippocampus during the postpartum phase, have not been fully explored [34].

In addition to hormones, it is also plausible that the environmental enrichment (pup exposure) contributes to the enhanced learning abilities of the mothers. However, it should be noted that the pups also serve as a source of stimulation for the control and pregnant females, given that they reside together in the IntelliCage with the mothers for an equivalent period. Consequently, a considerable proportion of the control females display sensitisation, exhibiting retrieval and licking of the pups despite not having given birth. Kinsley et al., observed that sensitised rats demonstrated enhanced performance on a reference memory task relative to nulliparous, but not primiparous rats (with mothering experience) following 16 days of pup exposure [24]. As compared to spatial tasks, executive functions like cognitive flexibility during pregnancy and motherhood in animal models have been less thoroughly studied. The postpartum period was associated with an enhanced performance in only the most complex extra-dimensional set shifting component of the tasks. This enhanced cognitive flexibility was associated with increased spine density in the prefrontal cortex [28]. This finding is somewhat at odds with our data, which demonstrates a more general improvement of cognitive capability in the postpartum period. This discrepancy could be attributed to the use of the drinking behavior associated with the motivational drive of the postpartum mothers. Executive functions were also found enhanced during late pregnancy and the early postpartum period, but this improvement faded after weaning [5]. This effect required oxytocin signaling and the presence of the offspring. Blocking oxytocin receptors in the prefrontal cortex or removing the pups negates the positive impact of motherhood on cognitive flexibility. However, after weaning, motherhood no longer enhances performance on tasks involving set-shifting, even in rats that have gone through one or multiple pregnancies [2].

## Conclusions

In summary, the present study provides compelling evidence that the postpartum period in female mice is associated with pronounced enhancements in spatial learning, cognitive flexibility, and temporal anticipation, as demonstrated using the IntelliCage system. These findings build on previous observations by employing a long-term, minimally invasive, and ecologically valid monitoring method, offering novel insights into the cognitive adaptations of motherhood. The enhanced performance of the postpartum females across multiple tasks is indicative not only of heightened motivation but also of underlying neurobiological changes, likely driven by the interplay of hormonal fluctuations and environmental factors such as pup interaction. These enhancements, particularly in hippocampus- and prefrontal cortex-dependent functions, may confer evolutionary advantages by improving a mother’s ability to navigate her environment and respond effectively to the dynamic demands of caregiving. However, a limitation of this study is that what is referred to as complex cognitive behavior cannot be completely divorced from simple drinking behavior. However, the finding that increased maternal drinking is primarily a consequence of an increased licking rate, while total nosepokes (a measure of appetitive drive) remain relatively unchanged during place learning, suggests that the increased place learning performance in mothers is a consequence of their elevated cognitive abilities. Therefore, this research highlights the importance of investigating the neuroendocrine and experiential mechanisms that shape maternal brain plasticity, with potential implications for understanding human maternal behavior and identifying novel targets for addressing cognitive impairments across clinical populations.

## Data Availability

The data presented in this study are available in the article, or are provided by the authors upon request.
